# Durable responses to ATR inhibition with ceralasertib in tumors with genomic defects and high inflammation

**DOI:** 10.1172/JCI175369

**Published:** 2024-01-16

**Authors:** Magnus T. Dillon, Jeane Guevara, Kabir Mohammed, Emmanuel C. Patin, Simon A. Smith, Emma Dean, Gemma N. Jones, Sophie E. Willis, Marcella Petrone, Carlos Silva, Khin Thway, Catey Bunce, Ioannis Roxanis, Pablo Nenclares, Anna Wilkins, Martin McLaughlin, Adoracion Jayme-Laiche, Sarah Benafif, Georgios Nintos, Vineet Kwatra, Lorna Grove, David Mansfield, Paula Proszek, Philip Martin, Luiza Moore, Karen E. Swales, Udai Banerji, Mark P. Saunders, James Spicer, Martin D. Forster, Kevin J. Harrington

**Affiliations:** 1 The Institute of Cancer Research, London, United Kingdom.; 2 The Royal Marsden NHS Foundation Trust, London, United Kingdom.; 3 Oncology R&D, AstraZeneca, Cambridge, United Kingdom.; 4 Clinical Pharmacology and Quantitative Pharmacology, Clinical Pharmacology and Safety Sciences, R&D, AstraZeneca, Cambridge, United Kingdom.; 5 UCL Cancer Institute and University College London Hospital NHS Foundation Trust, London, United Kingdom.; 6 King’s College London, and Guy’s and St. Thomas’ NHS Foundation Trust, London, United Kingdom.; 7 Oncology R&D, AstraZeneca, Gaithersburg, Maryland, USA.; 8 The Christie NHS Foundation Trust, Manchester, United Kingdom.

**Keywords:** Oncology, Cancer immunotherapy, DNA repair, Drug therapy

## Abstract

**BACKGROUND:**

Phase 1 study of ATRinhibition alone or with radiation therapy (PATRIOT) was a first-in-human phase I study of the oral ATR (ataxia telangiectasia and Rad3-related) inhibitor ceralasertib (AZD6738) in advanced solid tumors.

**METHODS:**

The primary objective was safety. Secondary objectives included assessment of antitumor responses and pharmacokinetic (PK) and pharmacodynamic (PD) studies. Sixty-seven patients received 20–240 mg ceralasertib BD continuously or intermittently (14 of a 28-day cycle).

**RESULTS:**

Intermittent dosing was better tolerated than continuous, which was associated with dose-limiting hematological toxicity. The recommended phase 2 dose of ceralasertib was 160 mg twice daily for 2 weeks in a 4-weekly cycle. Modulation of target and increased DNA damage were identified in tumor and surrogate PD. There were 5 (8%) confirmed partial responses (PRs) (40–240 mg BD), 34 (52%) stable disease (SD), including 1 unconfirmed PR, and 27 (41%) progressive disease. Durable responses were seen in tumors with loss of AT-rich interactive domain-containing protein 1A (ARID1A) and DNA damage–response defects. Treatment-modulated tumor and systemic immune markers and responding tumors were more immune inflamed than nonresponding.

**CONCLUSION:**

Ceralasertib monotherapy was tolerated at 160 mg BD intermittently and associated with antitumor activity.

**TRIAL REGISTRATION:**

Clinicaltrials.gov: NCT02223923, EudraCT: 2013-003994-84.

**FUNDING:**

Cancer Research UK, AstraZeneca, UK Department of Health (National Institute for Health Research), Rosetrees Trust, Experimental Cancer Medicine Centre.

## Abstract 2

The PATRIOT in-human study shows the ATR inhibitor Ceralasertib is tolerated and associates with anti-tumor activity.

## Introduction

ATR (ataxia telangiectasia and Rad3-related) is a critical kinase in the DNA damage response (DDR) (1, 2). Preclinical data have identified multiple cancer-related phenotypes sensitizing tumor cells to monotherapy ATR inhibition (ATRi) (3). Additionally, ATRi potentiates DNA-damaging therapies, including chemotherapy, radiotherapy (4), and targeted therapies such as poly ADP-ribose polymerase (PARP) inhibitors (5), making it a promising combination partner. Emerging evidence suggests ATRi may also modulate antitumor immune responses (6–8).

**Figure 1:**
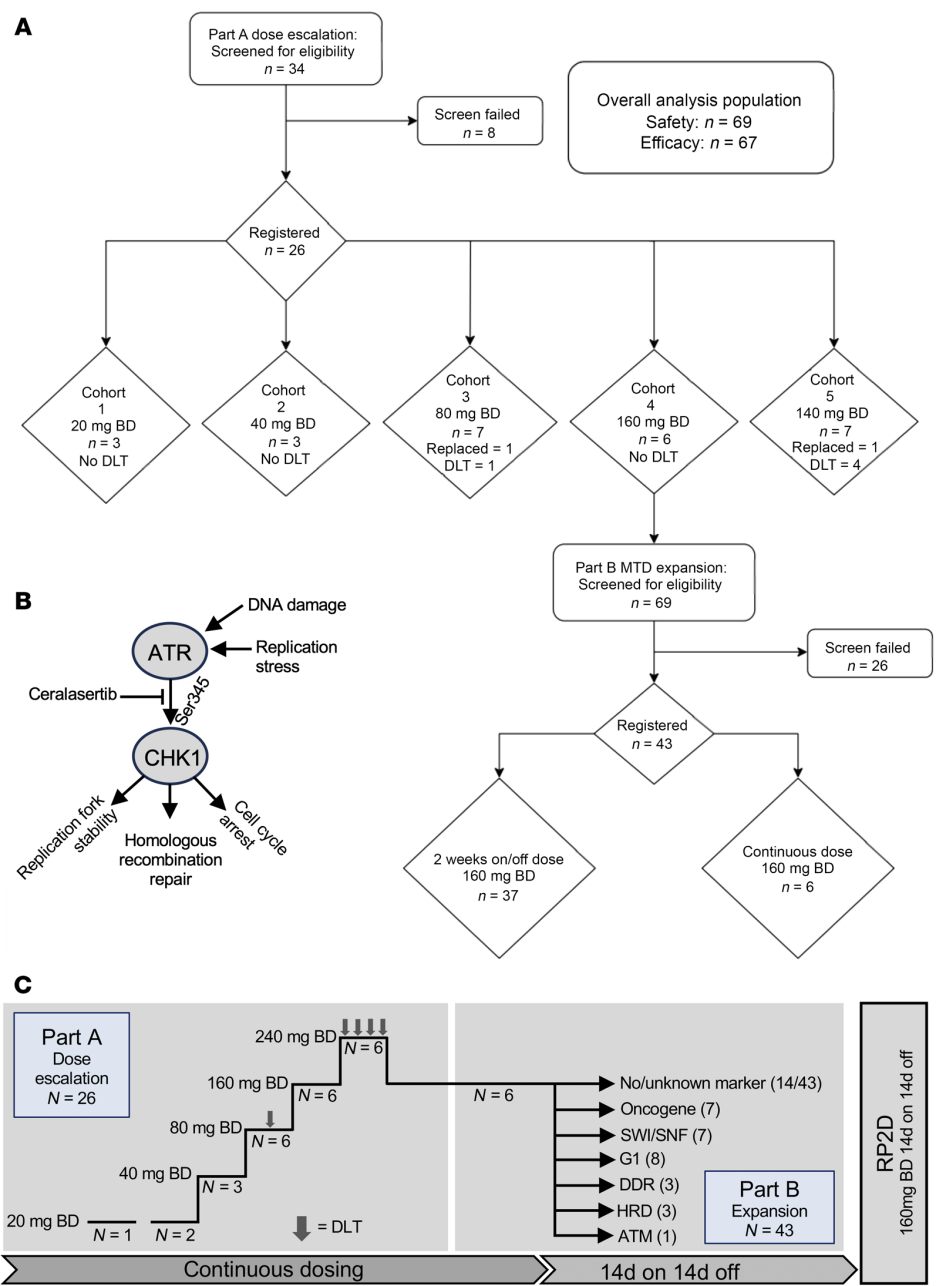
Study design. (**A**) CONSORT diagram of study parts A and B. (**B**) Schematic of role of ATR in DDR signaling. (**C**) Study schema for parts A and B. For part A, all patients received continuous dosing. For part B, they received continuous or intermittent dosing. Part B patients had mandatory tumor biopsy at baseline. All patients had PD sampling (PBMC, hair follicles) at baseline and at days 14–22. Response assessment was after 2 cycles of treatment.

ATR is activated by diverse DNA lesions causing exposure of expanses of single-stranded DNA (2). This replication stress is a frequent consequence of oncogene activation and impaired G1 checkpoint control and can be secondary to exogenous and endogenous sources of DNA damage and repair. Activated ATR phosphorylates targets, including checkpoint kinase 1 (Chk1), leading to stabilization of replication forks, activation of DNA repair, and activation of cell-cycle checkpoints (Figure 1B). Hence, monotherapy ATRi is predicted to affect tumors with high levels of replication stress, reduced DNA repair, or nonfunctional cell-cycle checkpoints, leading to accumulation of DNA damage and cell death.

In preclinical models, ATRi kills tumor cells with loss of ataxia telangiectasia mutated (ATM) (9), AT-rich interactive domain-containing protein 1A (ARID1A) (10), and specific components of the DDR pathway (11–13) or those driven by oncogenes such as cyclin E and Myc (14–16). Emerging data suggest that increasing the DNA damage load in cells could promote an antitumor immune response, for example, through interferogenic nucleic acid–sensing pathways (17).

Ceralasertib (AZD6738, AstraZeneca) (18) is a potent, selective, orally bioavailable, ATP-competitive ATR inhibitor, with antitumor activity demonstrated in multiple preclinical models (19). We report the results of the phase 1 study of ATR inhibition alone or with radiation therapy (PATRIOT) study (20), a first-in-human dose-finding study that determined safety, tolerability, recommended dose and schedule, pharmacokinetics (PKs), and antitumor activity of ceralasertib monotherapy and explored potential predictive biomarkers of response to ATRi.

## Results

### Patient characteristics

A total of 26 patients were enrolled and started ceralasertib in the dose-escalation phase across 3 centers between July 2014 and July 2016. In the dose-expansion phase, 43 patients were enrolled, of whom 41 received at least 1 dose of study drug (2 progressed prior to treatment start) between December 2016 and October 2020 (Figure 1A). Patient and tumor characteristics are given in Table 1.

### Dose escalation and toxicity

**Figure 2:**
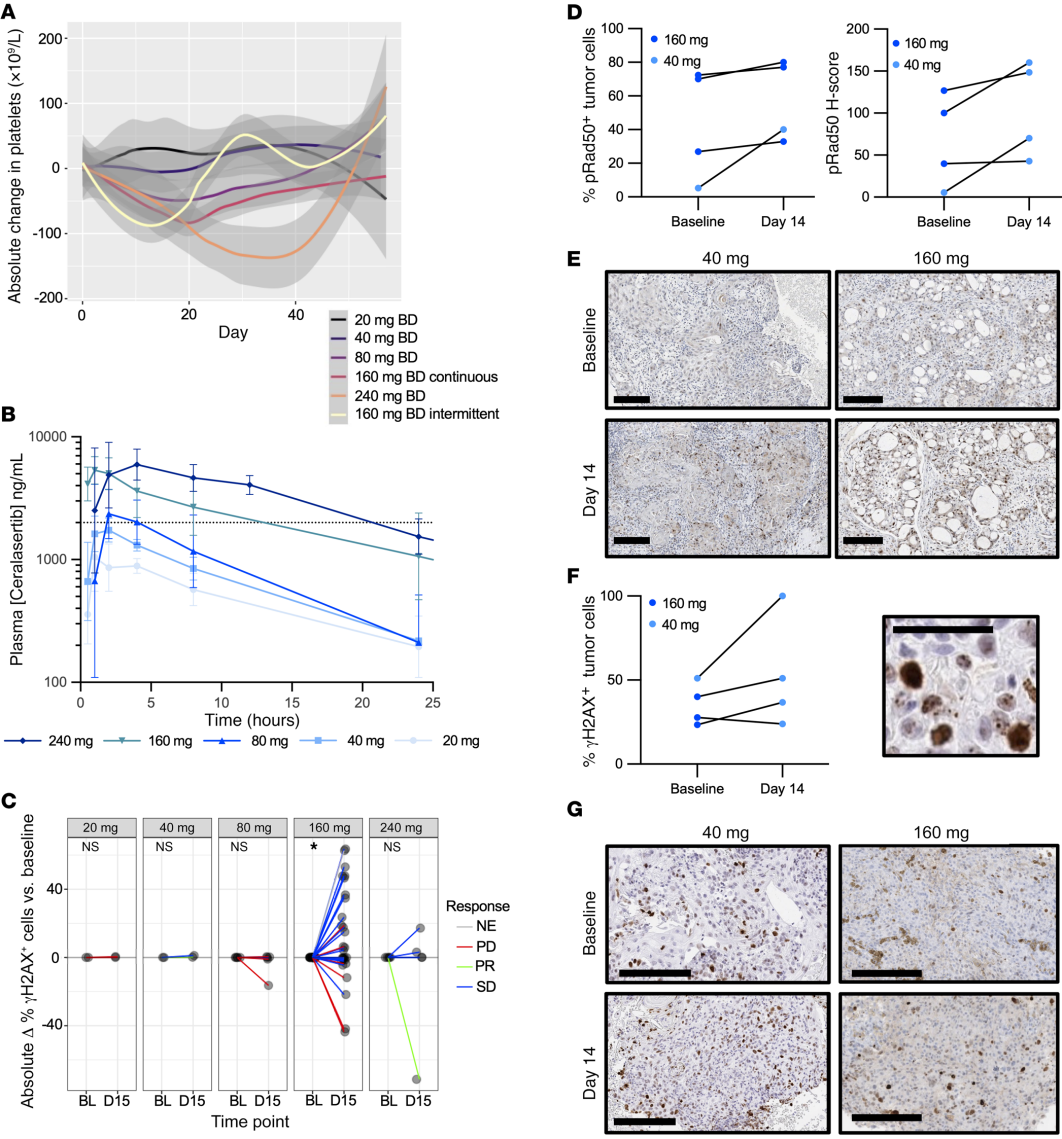
PKs and PDs. (**A**) Change in platelet count with time, by dose cohort. Smoothed conditional mean absolute changes compared with baseline blood count are presented, with 95% CI. (**B**) Ceralasertib PK. Geometric mean (and SD) plasma concentration over time after a single dose at the indicated dose levels (semi-log scale). (**C**) Absolute change in γH2AX-positive PBMCs (defined as percentage of cells with >5 foci) after 2-week dosing at the indicated dose levels. Line color indicates RECIST response. *P = 0.046, Wilcoxon’s signed rank test with a hypothetical median of 0. (**D**) Tumor PDs. Change in p-^(S635)^Rad50 in paired tumor biopsies after 2-week dosing. p-Rad50 in tumor cells expressed by percentage positive (left) and H score (right) for the indicated dose levels. Fold change versus baseline. *P* = 0.13, Wilcoxon’s signed rank test. (**E**) Examples of staining for p-Rad50 for the indicated dose levels. Scale bars: 200 μm. Left panel: HNSCC, 40 mg BD, RECIST PR. Right panel: parotid adenocarcinoma, 160 mg BD, RECIST SD. (**F**) Evidence of increased replication stress with ceralasertib treatment. Immunohistochemical staining for γH2AX in paired tumor biopsies. Left: change in percentage of positive tumor cells (defined as at least 5 nuclear foci or pan-nuclar staining) after 2-week dosing. *P* = 0.22, paired *t* test. Right: examples of nuclear foci and pan-nuclear staining after treatment. Scale bar: 50 μm. (**G**) Examples of γH2AX staining for the indicated dose levels. Left panel: HNSCC, 40 mg BD, RECIST PR. Right panel: serous ovarian carcinoma, 160 mg BD, RECIST SD. Scale bars: 200 μm.

A total of 67 patients received a dose of ceralasertib and were evaluable for safety (Figure 1A). Twenty-six patients were treated with continuous dosing schedule during the dose-escalation phase, at doses from 20 to 240 mg BD (Figure 1C). At the maximum administered dose of 240 mg BD, 3 of 6 patients had dose-limiting toxicities (DLTs). There were no DLTs at 160 mg BD and 1 at 80 mg BD (grade 3 [G3] thrombocytopenia with epistaxis, Table 2 and Supplemental Table 2; supplemental material available online with this article; https://doi.org/10.1172/JCI175369DS1). The maximum tolerated dose was 160 mg BD. DLTs were thrombocytopenia (G4, *n* = 2 at 240 mg, G3 with epistaxis, *n* = 1 at 80 mg) and elevated amylase (G3, *n* = 1 at 240 mg, Supplemental Table 2). Dose-expansion participants received 160 mg BD, either continuously or on a 2-week-on, 2-week-off schedule (Figure 1C). This was investigated after the development of toxicity beyond the DLT window in continuously dosed patients, leading to dose modifications. The intermittent schedule was chosen based on modeling of bone marrow recovery and was better tolerated, with incidence of G3 or greater anemia, 33% on continuous versus 9% on intermittent schedule, and 8% versus 0 % G3 leukopenia. Platelets and other hematological parameters were also more favorable with an intermittent schedule, recovering in the treatment break (Figure 2A, Table 2, and Supplemental Figure 1) (21). Six of 12 (50%) patients on the continuous schedule (including those in part A) versus 10 of 35 (29%) on the intermittent required dose reduction or interruption for toxicity. Four patients in the dose-escalation and 1 in the dose-expansion phase withdrew due to toxicity. There were no treatment-related deaths. Four deaths occurred on study medication: 2 from disease progression, 1 from pneumonia, and 1 from adult respiratory syndrome assumed to be COVID-19 related (no leukopenia observed for the latter 2 participants). The recommended phase 2 dose (RP2D) for the intermittent schedule was 160 mg BD, although other doses were not evaluated on an intermittent schedule. Serious adverse events related to study treatment are shown in Supplemental Table 1.

### PKs

Ceralasertib was rapidly orally absorbed across all doses following single and multiple dose administration (median time to peak drug concentration [t_max_] 0.5 to 4 hours), with mean terminal elimination half-life of 5.3 to 7.7 hours at the 40 and 80 mg dose levels and 11.2 to 12.8 hours at the 160 and 240 mg dose levels. Following single dosing, ceralasertib exposure increased approximately proportionally with increasing doses between 80 to 240 mg (Figure 2B). There was some evidence for accumulation after repeated dosing with higher predose and maximum concentration (C_max_) levels at days 15 and 29 compared with day 0. Accumulation ratios based on C_max_ and AUC were between 1.6- and 2.2-fold higher (Supplemental Figure 2).

### Pharmacodynamics

Paired PBMCs were available for the majority of study participants. PBMCs were analyzed for p-Chk1, the downstream phosphorylation target of ATR. There was variation in p-Chk1 levels with treatment, but this was not consistent (Supplemental Figure 3). p-Chk1 has been described as decreasing with ATRi in the presence of exogenous DNA damage (4) and as increasing with ATRi reflective of replication stress and DNA damage (22). Increased γH2AX positivity was observed in PBMCs after treatment at the RP2D for most subjects (Figure 2C), likely reflecting DNA damage in proliferating bone marrow cells due to ATRi. Four paired tumor biopsies were available for IHC. These tumor biopsies showed upregulation of p-Rad50, a marker of ATM pathway activation, after treatment with ceralasertib (Figure 2, D and E), as well as an increase in the number of γH2AX-positive cells (Figure 2, F and G).

### Response

At data cutoff, 4 patients remained on study; all had received a minimum of 24 cycles. Sixty-six patients were evaluable for response assessment, 26 in the dose-escalation and 40 in the dose-expansion phases.

**Figure 3:**
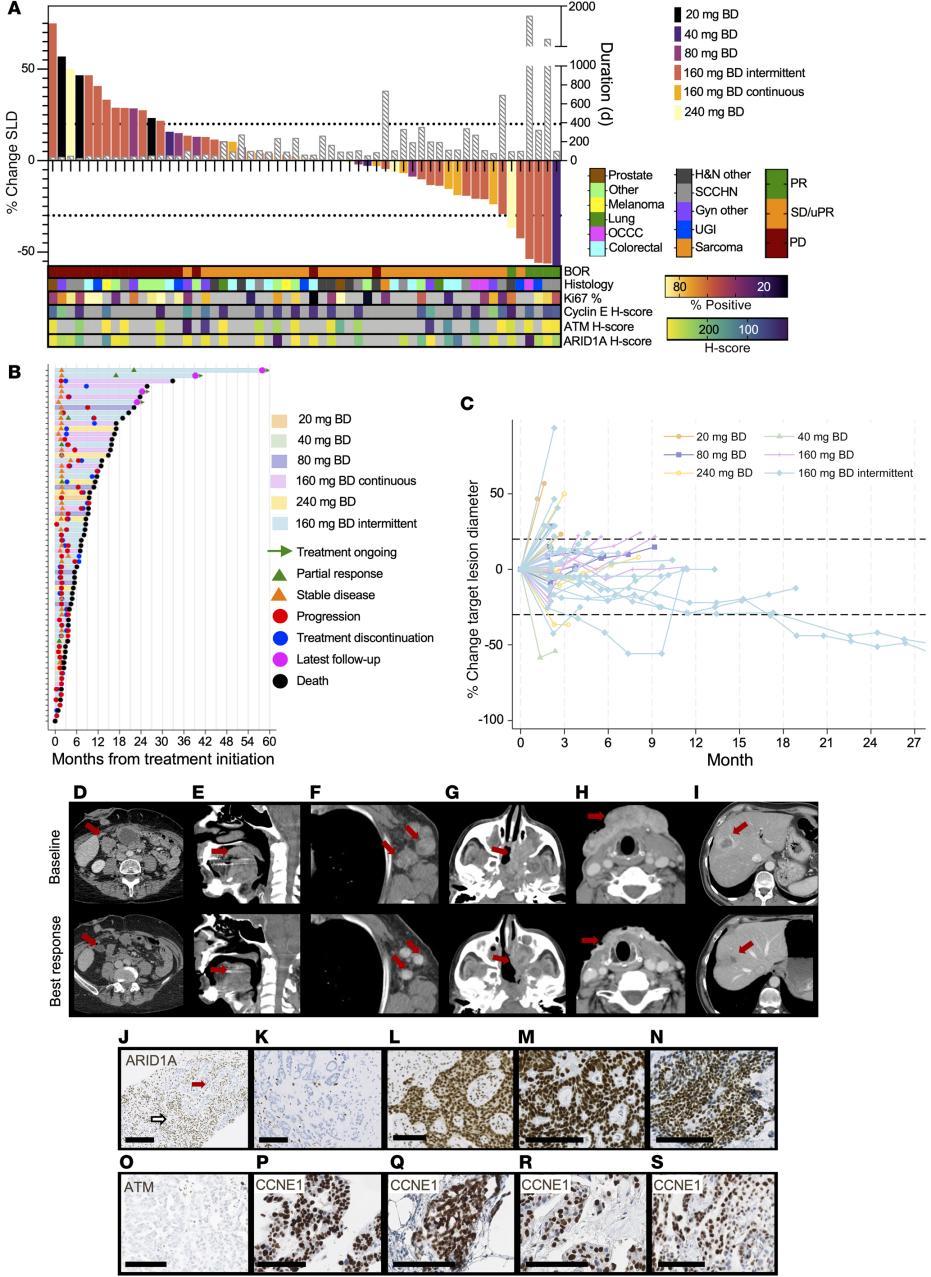
Antitumor responses. (**A**) Waterfall plot of best change in sum of longest diameters of target lesions (SLD), with corresponding duration on study. (**B**) Swimmer plot of evaluable patients. (**C**) Spider plot of evaluable patients. (**D**–**I**) Representative scans from responding patients. (**D**) Ovarian clear cell carcinoma, ARID1A loss, RECIST PR, 1,763 days on study, 160 mg BD, intermittent. (**E**) HNSCC, MRE11, and CDKN2A mutation, 1,194 days on study. (**F**) Esophageal squamous cell carcinoma, HR and Fanconi pathway deficiency, RECIST PR, 575 days on study, 160 mg BD intermittent. (**G**) Nasopharyngeal carcinoma, NRAS activation, RECIST PR, 341 days on study, 240 mg BD. (**H**) HNSCC, RECIST PR, 106 days on study, 40 mg BD. (**I**) Pancreatic adenocarcinoma, no clear mutation, unconfirmed PR, 480 days on study, 160 mg BD intermittent. Tumor protein profiling: IHC tumor staining was performed on the cases mentioned. Arrows indicate responding tumor lesions. (**J**) Clear cell ovarian carcinoma with loss of ARID1A, H score 0. Red arrowhead indicates tumor cells; white indicates stroma. (**K**) Eccrine adenocarcinoma with loss of ARID1A, H score 0. (**L**) Lung adenocarcinoma, ARID1A mutation but no protein loss. H score 290. (**M**) Cervix adenocarcinoma, ARID1A mutation but no protein loss. H score 300. (**N**) Clear cell ovarian carcinoma, ARID1A mutation but no protein loss. H score 235. (**O**) Serous ovarian carcinoma, ATM protein loss. (**P**) Same tumor as in **M**, showing cyclin E1 overexpression. H score 169. (**Q**) Peritoneal carcinoma, CCNE1 amplification on sequencing, cyclin E1. H score 210. (**R**) Serous endometrial carcinoma, CCNE1 amplification on sequencing, cyclin E1. H score 224. (**S**) Serous endometrial carcinoma, CCNE1 overexpression by IHC, cyclin E1. H score 155.

The best overall responses were 5 (8%) confirmed partial responses (PR), 34 (52%) stable disease (SD), including 1 unconfirmed PR, and 27 (41%) progressive disease, including clinical progression (Figure 3, A–C). Of those with SD or better, 25 of 39 (68%) had duration on study of at least 4 months, with many showing a slowing of tumor growth (Supplemental Figure 4). For those taking 160 mg BD or more, 4 of 49 (8%) had PR, 30 (61%) SD, and 15 (30%) progressive disease.

Patients with Response Evaluation Criteria in Solid Tumors (RECIST) (23) PR were dosed at 40, 240 (continuous schedule), and 160 (intermittent schedule) mg BD. Median duration of response was 46.7 weeks (IQR, 14.9–251.0). Responding histologies were as follows: (a) ovarian clear cell carcinoma with *ARID1A* mutation and high mutational load (160 mg BD, remains on study, 251 weeks at data cutoff; Figure 3D, Supplemental Table 4), (b) head and neck squamous cell carcinoma (HNSCC) with *CDKN2A* and *MRE11A* frameshift (160 mg BD, 170 weeks, remains on study; Figure 3E), (c) esophageal squamous cell carcinoma with homologous recombination (HR)/Fanconi pathway deficiency due to *BRIP1* frameshift mutation and *PALB2* deletion (160 mg BD, 47 weeks; Figure 3F), moderate mutational load (12.4 mutations/Mb), and APOBEC mutational signature (24–26); (d) nasopharyngeal carcinoma with *NRAS* activating mutation (240 mg BD, 14 weeks; Figure 3G), and (e) HNSCC with *APC* frameshift and *TP53* mutation (40 mg BD, 15 weeks; Figure 3H). One participant had an unconfirmed PR; this patient had *TP53* mutant pancreatic adenocarcinoma with no other mutation (160 mg BD, 15 weeks; Figure 3I). Patients with durable RECIST SD included those with HNSCC with *ARID2* frameshift (99 weeks), HNSCC with no sequencing available (48 weeks), HNSCC with *CCND1* amplification (49 weeks), and digital papillary adenocarcinoma with *TP53* mutation (51 weeks).

### Genomic and molecular correlates

Sequencing data were available for 5 of 26 patients in the dose-escalation and 36 of 41 in the dose-expansion phases. Patients with durable responses all had an alteration that may sensitize to ATRi (Supplemental Table 3 and Supplemental Figures 5 and 6). For patients dosed at 40 mg BD or more, out of 11 patients with no mutation of interest, 1 had a PR (9%) and out of 30 with a mutation of interest, 4 had a PR (13%). Of those with PR or SD, median duration of response was 105 days for those without a mutation of interest and 185.5 days for those with a mutation of interest. Unless otherwise stated, participants were taking 160 mg BD of intermittent ceralasertib.

### Durable responses in tumors with SWI/SNF loss

The most durable response was in a patient with clear cell ovarian carcinoma and an *ARID1A* mutation (*E21763fsX*) with loss of protein expression (Figure 3, D and J). Seven participants had aberrations in the SWI/SNF pathway, of whom 6 derived clinical benefit. One other patient had a clear ARID1A loss on IHC: a patient with eccrine adenocarcinoma with *ARID1A* stop-gain mutation (*R693X*, resulting in truncated protein expression) and H score of 0 (Figure 3K) with *CDKN2A* deletion (240 mg BD; SD, 34 weeks). A patient with an *ARID2* frameshift-bearing HNSCC had tumor shrinkage of 29% and remains on study at this writing after 99 weeks.

Other SWI/SNF aberrations are described in Supplemental Table 4. Notably, all other *ARID1A* mutants showed high protein expression (Figure 3, L–N), and the responding patient also had a high tumor mutational burden (TMB); there was no clear difference in TMB between patients with or without clinical benefit (Supplemental Figure 7).

### ATM pathway

There was no relationship between ATM expression and response or duration on study (Figure 3A). Twenty patients had ATM protein assessed: 4 were defined as ATM-low, with 25% or less ATM nuclear positivity (10%, 10%, 5%, and 0%; Figure 3O). Out of these, median duration on treatment was 13 weeks (range 8–29) with 3 of 4 experiencing SD and 1 progressive disease.

One patient had a pathogenic *ATM* mutation (*R1898fsX*) with some protein expression (50% nuclear positive) and a coexisting *ARID1A* mutation (see above), remaining on study for 39 weeks with SD. Another had *MRE11* stop-gain mutation (R633X), together with *CDKN2A* stop-gain, and remains on study at this writing after more than 32 months with a confirmed PR (Figure 3E). MRE11, a component of the MRN complex, activates ATM after DNA damage.

### Other aberrations

#### Oncogene amplification.

We identified 11 patients with oncogene-driven tumors (5 NRAS, 2 HRAS, 1 KRAS activation, 2 CCNE1 amplification, 1 CCND1 amplification), of whom 3 derived clinical benefit (Supplemental Figure 5). Of those with CCNE1 amplification, 1 (peritoneal carcinoma, 20 mg BD; Figure 3Q) had a best response of progressive disease, 1 (serous endometrial carcinoma; Figure 3R) SD, on study for 12 weeks, and 2 others had increased cyclin E1 expression by IHC without gene amplification: 1 with serous endometrial carcinoma (Figure 3S, with germline BRCA1 mutation) and the other with cervical adenocarcinoma (Figure 3P), both with SD for 16 and 29 weeks, respectively.

#### P53.

We have previously demonstrated no relationship between p53 functionality and ATRi sensitivity in a panel of cell lines (4). This was confirmed by the lack of difference in clinical benefit and duration on study between p53 WT and p53-mutant/p53-deleted tumors (Supplemental Figure 6).

### ATRi modulates the tumor-immune microenvironment

**Figure 4:**
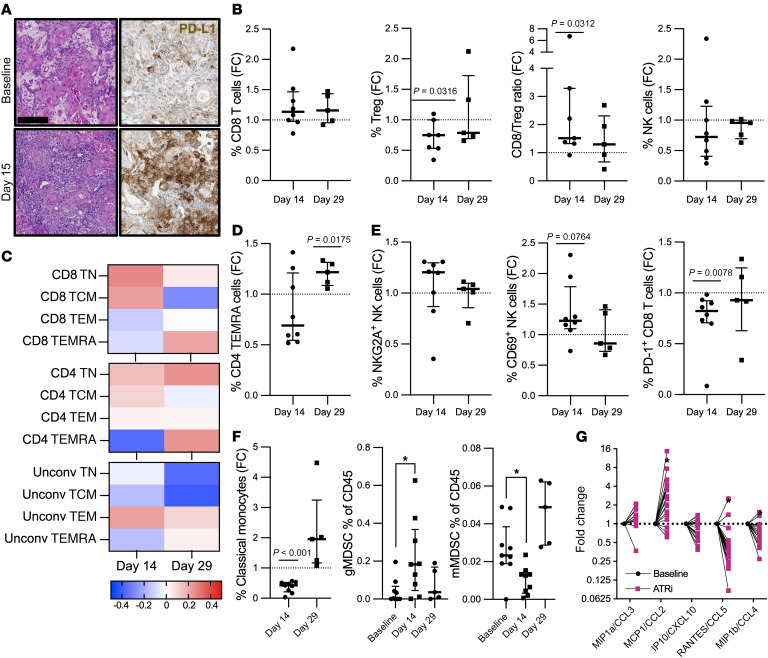
Immune profiling. (**A**) H&E and PD-L1 IHC staining of paired biopsies of a responding patient (HNSCC, 40 mg, RECIST PR), showing infiltration of PD-L1–positive immune cells after 2 weeks of ceralasertib. Scale bar: 200 mm. (**B**) Fold change (FC) in percentage of CD45^+^ cells in peripheral blood after 2 weeks of ceralasertib (day 14) and after a 2-week break (day 29) compared with baseline sample for the indicated cell type. Median and IQR indicated. Statistical significance by Wilcoxon’s test. (**C**) Shown is log_2_ fold change in percentages of the CD8^+^ T, CD4^+^ T, and unconventional (Unconv) T cells of the following populations: TN (T naive as CCR7^+^CD45RA^+^), TCM (T central memory as CCR7^+^CD45RA^–^), TEM (T effector memory as CCR7^–^CD45RA^–^), and TEMRA (T effector memory RA as CCR7^–^CD45RA^+^) from baseline, median, and IQR indicated. (**D**) Fold change in percentage of CD45 of memory CD4-TEMRA (effector memory reexpressing CD45RA) from baseline. Median and IQR indicated. Statistical significance by Wilcoxon’s test. (**E**) Fold change in percentage of NK cells or CD8^+^ T cells in the peripheral blood of (from left to right) NK cell NKG2A-positive, NK cell CD69-positive and CD8^+^ T cell PD-1–positive from baseline. Median and IQR indicated. Statistical significance by Wilcoxon’s test. (**F**) Left: fold change in percentage of CD45 of classical monocytes, as above. Middle: change in gMDSC as a percentage of CD45-positive cells, right: change in mMDSC as a percentage of CD45-positive cells. Median and IQR indicated. *P < 0.05, unpaired *t* test. (**G**) Fold change versus baseline in levels of the indicated plasma cytokines after 2 weeks of ceralasertib. *P < 0.05, paired *t* test.

We have previously shown preclinically that ATRi can affect the immune tumor microenvironment (TME), particularly when combined with radiotherapy (6, 27). In support of this, paired biopsies from a responding patient (40 mg BD, HNSCC, RECIST PR) showed an increase in immune-cell infiltration and programmed death-ligand 1 (PD-L1) staining on immune cells at 2 weeks (Figure 4A).

Therefore, we profiled, in detail, the immune response to ATRi in the peripheral blood of 8 participants (best responses of 5 SD, 2 progressive disease, and 1 nonevaluable [NE], all treated with 160 mg BD intermittent schedule) and in paired tumor biopsies (on treatment versus baseline) from 8 participants (5 SD, 2 progressive disease, and 1 NE). In the peripheral blood, we observed a reduction in Tregs and a trend toward increased CD8^+^ T cells, leading to an increased CD8/Treg ratio after ATRi (Figure 4B). All were on an intermittent schedule, allowing assessment of changes after 2 weeks of ceralasertib (day 14) and a 2-week break (day 29). Proportions of T cell subsets changed after ATRi, with increased naive and central memory CD8^+^ and CD4^+^ T cells after ATRi (Figure 4C). Importantly, there were increased frequencies of memory CD4-TEMRA (effector memory reexpressing CD45RA) cells at day 29 (Figure 4D). Detailed profiling revealed a reduction in PD-1–positive CD8^+^ T cells and NK cell activation, with a trend toward increased NKG2A- and CD69-positive NK cells with ceralasertib, which normalized after the 2-week break (Figure 4E). The circulating myeloid compartment was also altered by ATRi, with a reduction in classical and intermediate monocytes and a change in circulating myeloid-derived suppressor cells (MDSCs), with increased granulocytic MDSCs (gMDSC) and reduced monocytic MDSCs (mMDSCs) after ceralasertib, again trending to baseline after treatment break (Figure 4F). Circulating cytokine levels were modulated on ceralasertib therapy, with an increase in CCL2 and decrease in CCL4 and CCL5 levels observed after 2 weeks of treatment (Figure 4G).

### Responders to ATRi have inflamed tumors

**Figure 5:**
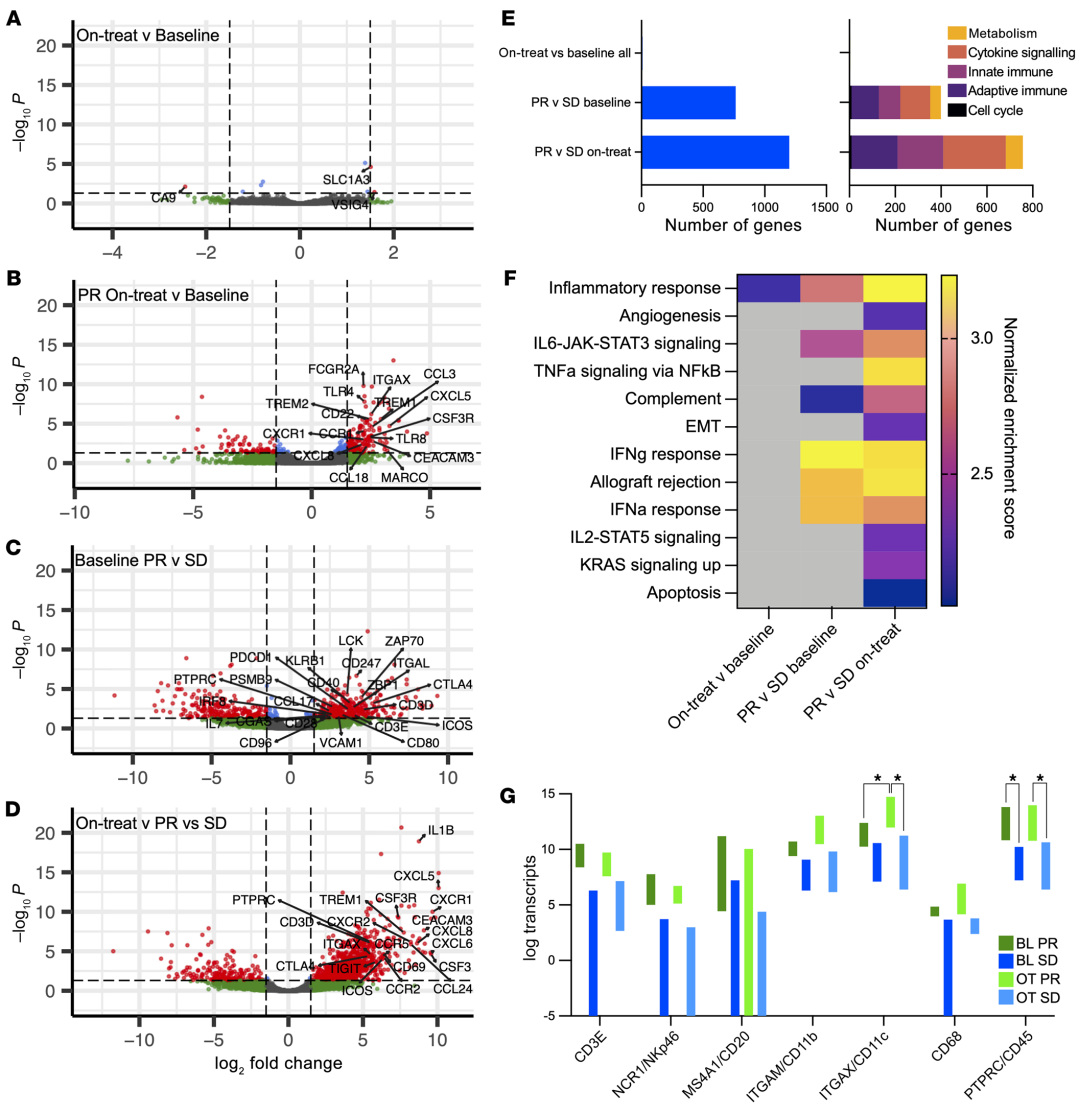
Tumor analysis. (**A**–**D**) Volcano plots of differential gene expression for the indicated conditions; log_2_ fold-change cutoff was set at 2 (1.5 for **A**) and *P* value at 0.05. Labeled genes are the most differentially expressed genes, which are also present in the REACTOME innate immune, adaptive immune, or immune system gene sets. (**A**) All samples, on treatment versus baseline; (**B**) on treatment versus baseline in responders; (**C**) responders versus nonresponders, baseline biopsies; (**D**) responders versus nonresponders, on-treatment biopsies. (**E**) Left: number of significantly differentially expressed genes from paired tumor RNA-Seq, for the indicated conditions. Right: number of genes in the indicated REACTOME pathways represented among differentially expressed genes for the indicated conditions (not all pathways are shown). (**F**) GSEA of tumor RNA-Seq data using the hallmarks gene set. For the indicated conditions, those pathways with normalized enrichment scores of more than 2 are shown. All have nominal *P* value and FDR *q* value of 0.000. OT, on treatment; BL, baseline. Heatmap indicates normalized enrichment score for the indicated gene sets. (**G**) Gene expression (minimum to maximum) for the indicated genes, in tumor biopsies at baseline and after 2 weeks of ceralasertib. *P < 0.05, 2-way ANOVA.

RNA-Seq of paired tumor biopsies was performed to assess differential gene expression after 2 weeks of ceralasertib treatment. Eight paired tumor biopsies were analyzed from 3 patients with PR, 4 with SD, and 1 NE, treated at various dose levels. Additional baseline samples were also available for 1 PR and 1 SD. When all samples were considered together, there were few differences in differential gene expression between baseline and on-treatment biopsies (Figure 5A). However, when responders (PR) were compared with nonresponders (SD), there were marked differences in both baseline and on-treatment gene expression (Figure 5, B–E) with clustering of a number of differentially expressed genes according to response (Supplemental Figure 8). The most common genes that were differentially expressed were immune related, with adaptive, innate, and cytokine-related genes highly represented (Figure 5E). Pathway analysis of the most differentially expressed genes found that these were predominantly immune related (Supplemental Figure 9). Gene-set enrichment analysis (GSEA) revealed enrichment of inflammatory response genes between baseline and on-treatment samples. When responders were compared with nonresponders, responding patients had more inflamed tumors both at baseline and on treatment, with significantly higher transcript levels for multiple immune-related genes (Figure 5F). Expression of cell-type–specific genes was different between responders and nonresponders. Responders had a significantly higher expression of *PTPRC* (CD45) at baseline and on treatment than nonresponders; they also had an increase in *ITGAX* (CD11c) with treatment. Several other genes showed similar elevation in responders compared with nonresponders, but this did not reach statistical significance (Figure 5G). When gene expression data were used for cell-type deconvolution, some differences were observed with treatment, particularly in neutrophil and macrophage populations (Supplemental Figure 10).

**Figure 6:**
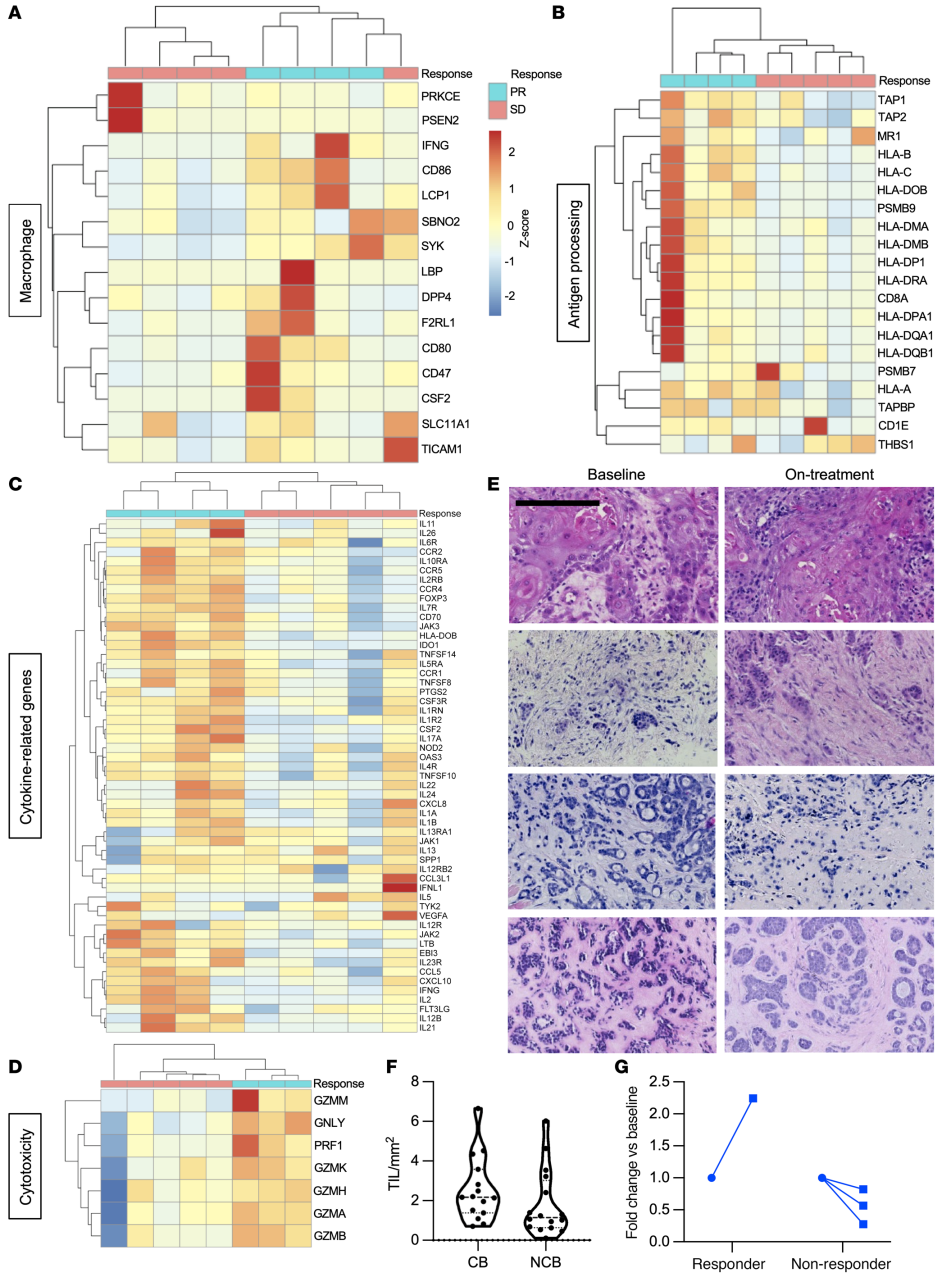
Tumor analysis. (**A**) Heatmap of macrophage-related gene expression in baseline tumor biopsies. (**B**) Heatmap of antigen processing–related transcripts in baseline biopsies. The first column represents the participant shown in Figure 2D, with high mutational burden. (**C**) Heatmap of cytokine-related gene expression in baseline tumor biopsies. Scale = *z* score, scaled by row. (**D**) Heatmap of cytotoxicity signature in on-treatment biopsies. (**E**) Representative images of tumor micrographs quantified in **G**. Top 3 rows: participants with SD. Lower row: participant with PR. Scale bar: 200 μm. (**F**) Stromal TIL count in H&E sections of patients who experienced clinical benefit (CB) (defined as PR or >16 weeks on study) compared with those who did not. (**G**) Fold change in stromal TILs in a responding patient and 2 nonresponding patients.

Baseline expression of macrophage, antigen-processing, and cytokine-related genes was generally higher in responding tumors (Figure 6, A–C), with clustering by response. T and NK cell signatures were increased in responders (Supplemental Figure 11, A and B). When on-treatment biopsies were analyzed, there was clustering of responders in cytotoxicity (Figure 6D) as well as cytokine and T cell signatures (Supplemental Figure 11, C and D). When plotted together, baseline and on-treatment samples tended to cluster by patient rather than by treatment, indicating a strong effect of baseline tumor inflammation on response. However, interferon-stimulated genes did appear to be upregulated in both baseline and on-treatment biopsies in responders (Supplemental Figure 12).

We counted stromal tumor-infiltrating lymphocytes (TILs) in H&E-stained sections at baseline and for 4 paired samples (Figure 6E). Those patients who derived clinical benefit from ceralasertib, defined as PR or greater than 16 weeks on study, had a trend to higher numbers of TILs than those who did not (Figure 6F). Stromal TILs appeared to increase in a responding patient, but not in 3 nonresponders (Figure 6, E and G).

## Discussion

Our study is the largest to date, to our knowledge, of ATRi monotherapy. We have shown that ceralasertib monotherapy is tolerable, with predominantly hematological toxicities reduced by an intermittent schedule. Ceralasertib has predictable PK and plasma levels at the RP2D that compare favorably with observed preclinical monotherapy IC_50_ values (ATR IC_90_ of 0.666 μM and GI_50_ of approximately 1 μM, comparable to between 270–420 ng/mL; refs. 4, 22). We have shown target modulation in tumor tissue and increased DNA damage in surrogate tissues. We found durable clinical benefit in diverse tumor types, with evidence suggesting multiple potential biomarkers of response to ATRi, including loss of ARID1A, genomic instability, ATM/G1 pathway abnormalities, and high tumor inflammation. Conversely, we did not find clear signals that oncogene drivers sensitize to ATRi.

Other published studies of ATRi have demonstrated similar, predominantly hematological, toxicities (28). ATRi has been combined with carboplatin (28) and with paclitaxel (29) in early phase studies. The only previously published ATRi monotherapy study of BAY1895344, to our knowledge, also found durable responses in DDR-defective tumors — 4 of 11 patients with ATM protein loss or deleterious mutation and 1 with BRCA1 mutation had prolonged SD (30). We identified several patients with ATM loss, all without objective responses. Responses were associated with other factors involved in G1 cell-cycle checkpoint control, including *MRE11*: loss will result in defective ATM activation, and a previous Chk1 inhibitor study observed a durable response associated with loss of another component of this complex (31).

Alternative ATR inhibitors are administered intravenously, and the duration of enzyme inhibition may differ between these 2 modes of administration. This may result in differential effects on efficacy and immunomodulation. As well as convenience, oral administration with an intermittent schedule allows bone marrow recovery between dosing periods and may allow more effective tailoring of dose exposure. The introduction of a modified schedule after emergence of toxicity outside the DLT window highlights a limitation of the 3+3 study design, and alternative designs may have been able to integrate such toxicities into dose-escalation decisions. Differences in efficacy between continuous, lower-dose and intermittent, higher-dose regimens should be examined in future studies.

ARID1A is a critical component of the SWI/SNF chromatin remodeling complex, which modulates the accessibility of DNA to transcription and repair machinery and is frequently mutated in cancers (32). ARID1A is important for ATR activation after double-stranded DNA breaks (33); it helps topoisomerase-II prevent DNA tangling during mitosis (decatenation) (34). Without this activity, cells activate a G2/M decatenation checkpoint (35). This is abolished by ATRi, leading to massive DNA damage (10). There are 2 main protein complexes in the SWI/SNF family: ARID1A, a critical component of the cBAF complex, and PBRM1 and ARID2, which are components of the PBAF complex (36). Only ARID1A loss has been described preclinically as an ATRi sensitizer (10), but distinct functions of different SWI/SNF complexes are unclear (36). ARID1A loss is particularly common in ovarian clear cell and uterine carcinomas (37). Of 2 patients with protein loss, 1 responded and 1 had SD, suggesting other factors may also be involved. Durable responses have been reported in patients with ARID1A loss in ongoing clinical studies (38, 39). Other components, such as ARID2, may also be associated with clinical benefit, as suggested by the durable SD in a participant with *ARID2* loss.

Intriguingly, ceralasertib responders in this study had more inflamed tumors at baseline. We saw ATRi-induced changes in the immune TME. Prior studies have found that ATRi can cause marked modulation of the TME, thought to be secondary to increased DNA damage and activation of cytoplasmic DNA-sensing machinery (6–8). Here, we have confirmed that treatment with ceralasertib modulates the immune response, with a more favorable CD8/Treg ratio, activation of NK cells, increased frequencies of effector memory RA CD4^+^ T cells, and modulation of cytokines and circulating MDSCs as well as increases in TILs and inflammatory gene expression in responding patients. A recently published combination study of ceralasertib and immune-checkpoint blockade (ICB) with durvalumab in advanced gastric cancer found a benefit in those patients with ATM loss or HR deficiency and found that responders had changes in their immune TME (40). However, the specific contribution of ATRi cannot be determined from those data. Our study of ATRi monotherapy allows an opportunity to observe the immunomodulatory effects of these agents without immunotherapies and provides the first data, to our knowledge, showing that ATRi (and other DDR inhibitors) may modulate the immune TME in their own right.

The possibility that inflamed tumors may respond better to ATRi suggests that (a) these tumors have preexisting DDR defects that make them more inflamed and more likely to respond to ATRi (41), with the inflammation being a phenomenon independent of the response to ATRi; and/or (b) there is modulation of antitumor immunity by the administration of ATRi. Although we noted that there may be increased TILs in the tumors of patients who benefitted from ATRi, the difference was modest and more substantial changes were seen between responders and nonresponders on the gene expression level. Notably, ATRi seem to increase responses to ICB in patients who have previously failed ICB alone (42), adding further weight to our hypothesis that ATRis have independent immunomodulatory effects. We suggest immune analyses in ongoing ATRi studies focusing on both baseline immune status and changes with therapy to uncover rational immunotherapy partners for ATRi. In particular, the effect we have observed on NK and myeloid cells should be further investigated, particularly in light of preclinical data suggesting NK cells may have a role in ATRi responses (27).

The results from this study provide the first evidence, to our knowledge, that ceralasertib monotherapy is tolerable, with antitumor activity in a number of genetic backgrounds. We have recommended a 160 mg BD 2-week-on, 2-week-off dosing schedule for further evaluation. Phase I–III studies are proceeding as monotherapy or in combination with poly (ADP-ribose) polymerase (PARP) inhibitors in advanced solid tumors (ClinicalTrials.gov NCT02264678), *ATM*- or *ARID1A*-mutant tumors (43) (NCT03682289), DDR-deficient tumors (NCT03462342), and in combination with ICB (44, 45) (NCT02664935, NCT05061134, NCT05450692). Tumor inflammation, ARID1A loss, and genome instability are among the most promising areas for future study.

## Methods

### Patient population.

Patients were 18 years and over, with advanced solid malignancy, without standard anticancer treatment options. All had Eastern Cooperative Oncology Group (ECOG) (https://ecog-acrin.org/resources/ecog-performance-status/) performance status 0–1, life expectancy of at least 3 months, and adequate organ function. Key inclusion criteria are provided in the Supplemental Methods.

### Study design.

This was a multipart, multicenter, open-label phase I study. Part A comprised a dose escalation and part B a dose expansion. During dose expansion, participants were selected based on the presence or absence of putative biomarkers of response to ATRi. Part C (combination with radiotherapy) will be reported separately. Patients in this study started ceralasertib between July 2014 and October 2020. The data cutoff was in October 2022, when 4 patients were still on study medication, all for at least 2 years.

The primary objective was to determine the safety and feasibility of administration of ceralasertib monotherapy in patients with advanced solid tumors. The secondary objectives were to identify a dose and schedule for further studies of ceralasertib and to assess antitumor responses and PK. Exploratory objectives included PD studies in tumor and normal tissue and the potential value of putative markers of sensitivity to single-agent ATRi, including measures of immune activation.

### Study treatments.

Ceralasertib was administered orally, twice daily.

For part A (dose escalation; Figure 1, A and B), the starting dose of 20 mg was selected based on animal toxicity studies. Dosing was continuous, and escalation used a modified Fibonacci method. Initial dose escalation was planned in single patient cohorts, changing to 3+3 design after the first grade-2 toxicity was seen. This occurred in the first patient. Cohorts of 3 to 6 patients were assessed for toxicity during a DLT window of 28 days (1 cycle), with a nontolerable dose defined as 2 or more of 6 patients experiencing a DLT. DLT definitions are in the Supplemental Methods.

For part B, all patients received the RP2D as defined for part A. Part B allowed for different schedules (continuous/intermittent) to be assessed. Initially, a continuous dosing schedule was used for the first 6 patients. Subsequently, the safety review committee authorized the assessment of an intermittent schedule, 14 days on and 14 days off. Pretreatment biopsy was mandatory in part B. DNA-Seq of archival tumor material or review of external tumor sequencing was used to enrich for patients with putative genomic markers of sensitivity to ATRi, based on preclinical data, including oncogene amplification or driver mutation, ATM/G1 pathway defects (alteration in ATM, CHK2, or other components of the pathway causing G1 cell-cycle arrest after DNA damage), SWI/SNF (switch/sucrose nonfermentable, a chromatin-remodeling complex) pathway defect, genomic instability/homologous recombination deficit (HRD), or defect in a gene synthetically lethal with ATRi in published data (46, 47) (Figure 1B and Supplemental Table 3).

### Study assessments.

Patients were assessed weekly during cycle 1 and twice weekly thereafter, with safety assessments including blood hematology and biochemistry, physical examination, and toxicity scoring. Safety and tolerability were assessed using Common Terminology Criteria for Adverse Events (CTCAE), version 4.03. Participants had ECG and urinalysis at the start of each cycle of treatment and assessment of left ventricular ejection fraction every 8 weeks. Response assessment imaging was conducted according to RECIST 1.1 within 28 days of starting ceralasertib and every 8 weeks.

### PKs.

Intensive PK sampling in part A occurred after a single dose from predose up to 24 to 72 hours and again at day 15 and day 29 of continuous dosing. Participants fasted for 1 hour before and 2 hours after dosing for PK assessment. In part B, sampling coincided with day 15 PD assessments. Full details are given in Supplemental Methods.

### PDs and histology.

PD sampling took place at baseline (within 7 days prior to dosing) and between days 15 and 22 of dosing (day 14 for intermittent dosing cohorts). PD samples included PBMCs and tumor biopsies. PBMCs were analyzed by immunofluorescence for γ^(S139)^H2AX, p-^(S345)^Chk1, and total Chk1, as described in Supplemental Methods. Paired tumor biopsies were formalin fixed and analyzed for nuclear p-^(S635)^Rad50 and γH2AX by IHC.

### Translational methods.

DNA-Seq of tumor and matched buffy coats was either by whole-exome sequencing or a custom-designed panel targeting all exons of genes of interest for 173 genes, including potential markers of sensitivity to ATRi. ACK-lysed whole blood or PBMCs were stained for flow cytometry using 8 multicolor panels. Plasma cytokines were assessed using the Bio-Plex Pro 27-Plex Panel (Bio-Rad). See Supplemental Methods for full information.

### Statistics.

Simple descriptive statistical data analysis methods were used to summarize the data. Categorical data used numbers and percentages of patients in the categories/groups; where appropriate, 95% CIs were reported. Continuous nonnormally distributed data as assessed by visual inspection were described using median, IQR, and minimum and maximum values. Statistical analyses were conducted using STATA, version 17.0. Additional genomic and laboratory data were plotted and analyzed using Prism 8 (GraphPad), and ggplot2 in R version 4. For comparison of biomarkers at baseline and on treatment, paired *t* tests (2 tailed) or their nonparametric equivalents were used. When comparing fold-change data normalized to baseline, Wilcoxon’s signed rank test with a hypothetical median of 1 (no change from baseline) was used unless otherwise stated.

### Study approval.

This study was conducted in accordance with protocol requirements, good clinical practice (GCP), and the Declaration of Helsinki. All participants provided written, informed consent. The protocol was approved by the local ethics committee (NRES Committee London–City and East, reference 14/LO/0465).

### Data availability.

Deidentified individual participant data that underlie the figures in this article will be made available to researchers who provide a methodologically sound proposal and complete the data access agreement. Tumor profiling, flow cytometry, and clinical annotations can be provided. PK and PD data cannot be provided. Values for all data points in graphs are reported in the Supporting Data Values file.

## Author contributions

Study conceptualization, design and protocol writing were by MTD, SAS, MDF and KJH. Study safety review was by KJH, UB, MPS, JS, and MDF. JG was the study manager and KM the statistician. PP and KES were responsible for genomic and PD assessments. IHC staining and analysis were supported by GNJ, SEW, KT, LM, PM, IR, and AW. PK analysis was performed by MP and CS. Immune assays and analysis were performed by ECP, MM, and DM. Authors contributing through participant support and site-level investigation were MTD, JG, ECP, GNJ, SEW, MP, CS, KT, IR, PN, AW, MM, AJL, SB, GN, VK, LG, MPS, PP, PM, LM, JS, MDF, and KJH. The original draft of the manuscript was written by MTD, KM, and KJH, and it was reviewed and edited by MTD, JG, KM, ECP, SAS, ED, GNJ, MP, CS, CB, PN, AW, PP, KES, UB, MPS, JS, MDF and KJH. All authors approved the final manuscript.

## Supplementary Material

Supplemental data

ICMJE disclosure forms

Supporting data values

## Figures and Tables

**Table 1:**
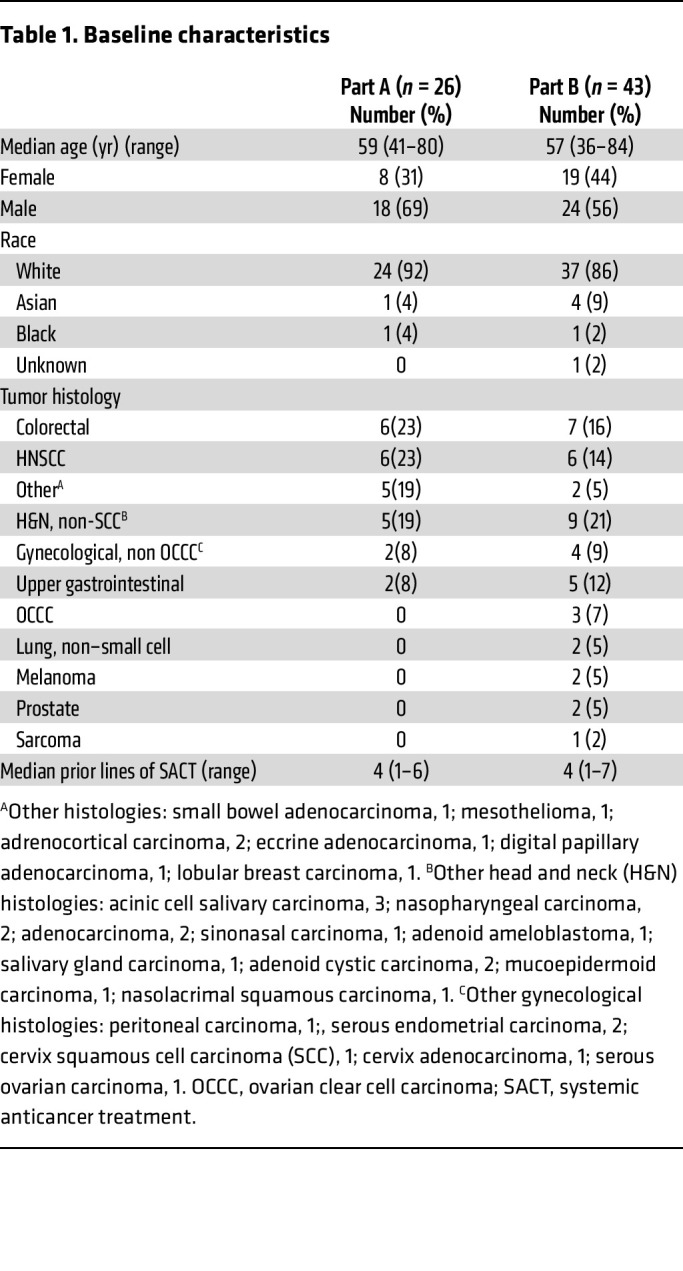
Baseline characteristics

**Table 2:**
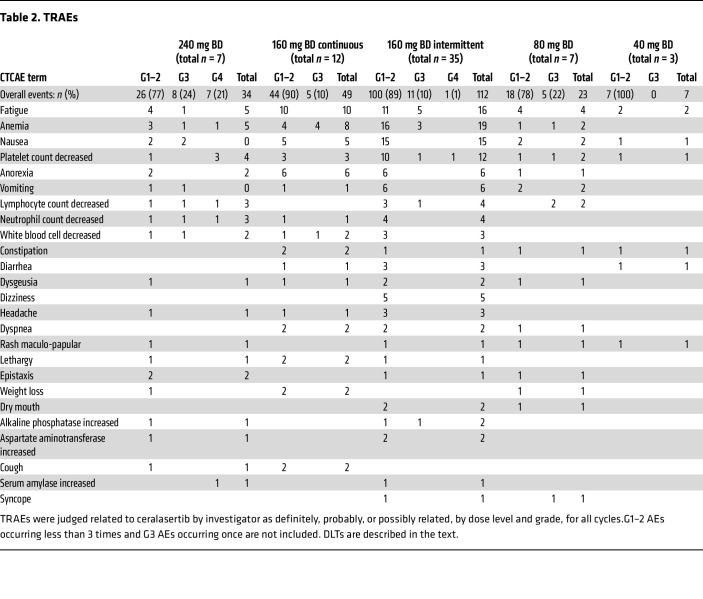
TRAEs

## Version 1 11/07/2023

In-Press Preview

## Version 2 01/16/2024

Electronic publication
