# Supplementary material for: Durable responses to ATR inhibition with ceralasertib in tumors with genomic defects and high inflammation
Source: J Clin Invest. 2024 Jan 16;134(2):e175369. doi: 10.1172/JCI175369 (PMC10786692; doi:10.1172/JCI175369)
Supplement: Supplemental data [file jci-134-175369-s115.pdf]

Supplementary materials and methods

Key inclusion and exclusion criteria

1. Part B only: documented disease progression prior to study entry and measurable disease by RECIST 1.1 within 4 weeks of study entry
2. Presence of putative markers of sensitivity to AZD6738 defined on analysis of tumor material (for part B only).
3. Evidence of measurable or evaluable disease by RECIST 1.1
4. Patients must have normal organ and bone marrow function measured within 7 days prior to administration of study treatment as defined below:
    - Hemoglobin ≥9.0 g/dL
    - Absolute neutrophil count (ANC) ≥ 1.5 x 10^9/L
    - White blood cells (WBC) > 3x10^9/L
    - Platelet count ≥ 100 x 10^9/L
    - Albumin >30g/L
    - AST and ALT <3 times ULN
    - Total bilirubin <1.5 times ULN
    - PT/APTT (<1.5 x upper limit of normal)
    - INR < 1.5 and no other evidence of impaired hepatic synthetic function
    - Glomerular filtration rate (GFR) >50mL/min, as assessed using the standard methodology at the investigating center (i.e., Cockcroft-Gault, MDRD or CKD-EPI formulae, EDTA clearance or 24-hour urine collection)
    - Serum creatinine <1.5 times ULN
    - Negative serum pregnancy test for females of childbearing potential
5. Part B only: tumor site amenable to fresh biopsy (clinical or radiologically- guided)
6. Not Receiving, or having received, concomitant medications, herbal supplements and/or foods that significantly modulate CYP3A4 or P-gp activity (wash out periods of two weeks, but three weeks for St. John’s Wort).

7. Not pregnant or breast-feeding, not of childbearing potential unless adequate contraception is used.
8. No clinically significant cardiac disease including:
    - pre-existing arrhythmia,
    - Any factor increasing the risk of QTc prolongation or arrhythmia,
    - uncontrolled angina pectoris,
    - Myocardial infarction 1 year prior to study entry,
    - Cardiac failure
9. No symptomatic and progressive or steroid-requiring brain metastases or leptomeningeal disease
10. Not hypertensive (clinically uncontrolled, or requiring 2 or more antihypertensive agents)
11. No hypotension or orthostatic hypotension
12. No hematuria (+++ on microscopy or dipstick)

Pharmacokinetics

Plasma samples (50 μL) were analyzed using validated bioanalytical methods for ceralasertib and its metabolite AZ13368982, after the addition of deuterated internal standards, by protein precipitation followed by reversed-phase high-performance liquid chromatography with tandem mass spectrometric detection (HPLC-MS/MS)._Concentrations of each analyte were calculated with reference to a calibration series covering the concentration ranges 41.3 to 41,300 ng/mL and 3.98 to 3,980 ng/mL for ceralasertib and AZ13368982, respectively, constructed by adding known amounts of each to control human plasma and processing these standards in parallel with the trial samples. Both pre- and in-study validation was successfully conducted according to the FDA's Guidance for Industry Bioanalytical Method Validation (47). Evaluated PK parameters for ceralasertib included area under the plasma concentration–time curve (AUC), maximum observed plasma concentration (C_{max}), time to C_{max} (t_{max}), and apparent terminal half-life ($t_{1/2}$) calculated as ln/lambda-z where lambda-z (λz) is the apparent terminal phase rate-

constant estimated by linear regression of logarithmically-transformed concentration-versus-time data. A minimum of three data points were used in calculating λz as per AstraZeneca standard operating procedures. Data from multiple dosing were used to derive accumulation ratios based on C_{max} (R_{ace} C_{max}) and AUC(0-8) (R_{ac} AUC_{0-8}) defined as the ratio between C_{max} or AUC(0-8) after multiple dose and C_{max} and AUC(0-8) at day 1. All PK parameters for ceralasertib were derived using non-compartmental analysis (NCA) method in Phoenix WinNonLin v8.3 software or higher where the 'linear up/log down trapezoidal rule' for AUC was applied. All PK concentrations and parameters were listed and summarized as per AstraZeneca standard operating procedures by dose level and by treatment (single or multiple dose).

Sequencing

Formalin-fixed samples were assessed by a pathologist for tumor-rich areas, which were marked for microdissection at extraction, and tumor content estimated. Tumor and normal tissue (DNA extracted from buffy coats) were analyzed where possible (and for all non-archival biopsies) to remove germline variants.

Formalin-fixed paraffin embedded (FFPE) tissue slides, fresh frozen material, and frozen buffy coat samples were extracted for this study.

FFPE slides were reviewed for tumor content by a pathologist and tumor rich areas were marked for macrodissection at extraction. DNA was extracted using from five 10-micron sections of FFPE tumor samples using QIAamp DNA FFPE tissue kit (56404 QIAGEN).

FF samples were extracted using QIAamp DNA Mini kit (51304 QIAGEN).

Frozen buffy coat samples were extracted on Qiagen QIAsymphony SP instrument, using QIAsymphony DNA_Blood_400_V6_DSP protocol, and QIAsymphony DNA Midi Kit (931255, QIAGEN).

DNA was quantified using the Qubit dsDNA High Sensitivity Assay Kit with the Qubit 3.0 Fluorometer (Invitrogen, Carlsbad, CA).

NGS libraries were prepared from 25-400ng DNA using the KAPA HyperPlus Kit (Kapa Biosystems, Wilmington, MA, USA) and IDT UDI 8bp adapters (Integrated DNA Technologies, Coralville, USA), following the manufacturer's protocol, including dual-SPRI size selection of the libraries (250-450 bp). To optimize enrichment and reduce off-target capture, pooled, multiplexed, amplified pre-capture libraries (up to 20 samples per hybridization) were hybridized overnight using 1 μg of total DNA to a custom design of DNA baits complementary to the genomic regions of interest (NimbleGen SeqCap EZ library, Roche, Madison, WI, USA). Hybridized DNA was PCR amplified and products purified using AMPure XP beads (Beckman Coulter, Danvers, MA, USA) and quantified using Qubit dsDNA High Sensitivity Assay Kit with the Qubit 3.0 Fluorometer (Invitrogen, Carlsbad, CA), and High Sensitivity D1000 TapeStation (Agilent, Santa Clara, USA).

Samples were captured using a targeted capture panel (DDR panel) consisting of 173 genes, including multiple potential sensitizers to ATRi, such as DDR genes, and oncogenes (*Supplementary Table 3)*.

Sequencing was performed on a NextSeq (Illumina, San Diego, CA, USA) with 75 bp paired-end reads and v2 chemistry, or NovaSeq6000 with 100 or 150bp paired-end reads and v1 chemistry, according to the manufacturer's instructions.

Sequencing runs were analyzed using an in-house pipeline. For the demultiplexing, Illumina bcl2fastq was used to assign reads for each sample based on the sequencing of 8-bp unique dual indexes. The reads were aligned to the reference genome build GRCh37/Hg19 using Burrows-Wheeler Aligner (BWA-MEM), followed by the marking of PCR duplicates and calculation of various quality control (QC) metrics using Picard. Genome Analysis ToolKit (GATK) was used for realigning around known indels to improve indel calling and base quality

score recalibration for adjusting systematic errors made by the sequencer when estimating quality scores of each base call. HaplotypeCaller is used for variant calling in germline sample (limit of detection ~10%) and Mutect2 is used for tumor-normal paired somatic analysis (limit of detection ~5%). Mutect2 tumor only mode is used for tumor only somatic analysis (limit of detection ~5%). VCF files were then annotated using oncotator (for samples pre-2019) and Personal Cancer Genome Reporter (for post 2019 samples). The potential mutations identified by in-house pipeline were further checked manually on IGV. Copy number variant was estimated by generalizing the coverage expected for a copy of any given targeted region (i.e.an exon), taking the average coverage across all captured regions to estimate the average coverage of one targeted region. Any ratio below 0.5-fold was defined as a potential deletion, whereas a ratio above 2.4 was flagged as a potential amplification if 80% of the target regions had exceeded the thresholds. Manta and Pindel was used for the detection of structural variants including large indels, potential fusions and ITDs.

For whole exome sequencing, genomic DNA was extracted from Buffy Coat using the Qiagen DNeasy Blood & Tissue Kit, from Frozen Solid Tumors using the Qiagen All Prep DNA/RNA Micro Kit, and from FFPE samples using the Covaris truXTRAC FFPE DNA Kit.

All genomic DNA (200-1000ng) was fragmented to 200bp using a Covaris E Series and the resultant libraries were subjected to DNA Capture using SureSelect XT Human All Exon v5 or v6 kit (Agilent) following the manufacturer’s instructions.

Final libraries were quantified using qPCR and clustered at a molarity of 14.5 pM; sequencing was performed on an Illumina HiSeq 2500 using 2x101 cycles of version 2 RAPID SBS chemistry. Tumor samples were sequenced at 100-150x depth and germline samples at 40-60x. Tumor mutational burden was defined as total number of somatic mutations, including

synonymous mutations, divided by the library panel size. Tumor mutational burden was defined as high (≥20 mutations/Mb), intermediate (10-20) or low (<10).

In part B, sequencing of archival tumor material, or external sequencing reports were used to select some patients for the study. Out of 21 archival/external sequencing, confirmatory sequencing using fresh biopsies was performed in 20 tumors, and was concordant in 10 (in 2, the gene of interest was not included on the sequencing panel; in 3, confirmatory sequencing found other mutations of interest, in 5, confirmatory sequencing found no mutations of interest).

Tumor RNAseq

RNA samples were quality-controlled and sequenced by the ICR Genomics Facility. RNA polyA method was used for mRNA selection. Strand-specific libraries were generated using the NEB ultra II directional kit. Illumina paired-end libraries were sequenced on a NovaSeq (Illumina) using Novaseq chemistry acquiring 100bp paired-end (PE) reads. Bcl2fastq software (v2.2.20, Illumina) was used for converting the raw base calls to fastqs and to de-multiplex further the sequencing data. The PE fastq files were used for further analysis. The STAR alignment software (v.2.7.6a) was used to align reads to the reference genome (GRCh38). Once the reads were aligned, HTSeq-count (HTSeq v0.12.4) was used to count the number of reads mapping unambiguously to genomic features in each sample.

Differential expression analysis of the count data was performed in R using the Bioconductor package DESeq2 (v1.34.0). Dispersion estimations were corrected using the fdrtool (v1.2.17). Gene Set Enrichment and Pathway Analysis was carried out in R using DOSE (v3.20.1), pathview (v1.34.0) and clusterProfiler (v4.2.2) packages, and using GSEA (v4.3.2) (48). Volcano plots were generated using EnhancedVolcano v1.16.0. Heatmaps were constructed using the pheatmap package (v1.0.12) using euclidean clustering and scaling by row; gene

signatures for heatmap construction were used from MSigDB (49) and cell-type-related transcripts from NanoString annotations and Bindea et al (50). Cell-type deconvolution was performed using CIBERSORTx (51).

Supplementary Pharmacodynamic methods

IHC

Formalin fixed paraffin-embedded tumor samples were used. Nuclear phospho-$^{(S635)}$Rad50 (Cell Signaling 14223), using a previously published method (52). H-score was calculated using HALO image analysis (Indica Labs). Tumor samples were also stained for γH2AX (S139, Cell Signaling 9718) positivity by IHC. A positive nucleus was defined as one with at least 5 nuclear foci or pan-nuclear staining, resulting in a total percent positive score. Nuclear ATM (Abcam 32420) IHC staining was carried out on the Ventana autostainer, using DAB detection and assessed by H-score, but samples were only deemed acceptable if lymphocyte staining (internal control) was at least 2+ (moderate) intensity. Cyclin E1 (Invitrogen HE12) staining was carried out on the Ventana autostainer, using DAB detection and the H-score was quantified using HALO image analysis (Indica Labs). Ki67 (Mib-1, Dako M7240) was scored by percent nuclear positivity using a global unweighted method, scoring 4 areas of 100 cells. ARID1A IHC was performed as previously published (36), using EPR13501 antibody (Abcam) and scored using H-score for nuclear positivity. Quantification of tumor-infiltrating lymphocytes was done using a semi-automated method in QuPath (53). Scanned H&E sections were first segmented into tumor and stroma, using a random trees machine-learning classifier, for each tumor section. Nuclei were segmented using Stardist (54), then stromal nuclei were sorted by size (area $<20\ \mu m^2$) and circularity (>0.85), using small circular nuclei to approximate lymphocyte count.

Immunofluorescence

PBMCs were isolated from blood using BD Vacutainer® CPT™ Cell preparation tubes with sodium citrate (BD Biosciences, Wokingham, UK) and fixed with 4% formalin containing 1% Triton™ X-100 (Sigma-Aldrich, St. Louis, MO). PBMC cells were cytospun onto microscope slides and stained with anti-phospho-$^{(S345)}$Chk1 [133D3] (Cell Signaling Technology 2348), or anti-γH2A.X$^{(S139)}$ (Abcam ab11174) antibodies and AlexaFluor 488 goat anti-rabbit IgG antibody (Invitrogen, Carlsbad, CA). Nuclei of PBMCs were counterstained with TOPRO-3 (Invitrogen). A Carl Zeiss LSM 700 confocal laser scanning fluorescence microscope (Zeiss, Jena, Germany) was used to visualize and capture images of the PBMCs. Fluorescent nuclear intensity for phospho-$^{(S345)}$ and total Chk1 in individual PBMC cells were quantified using the IN Cell Investigator Developer Toolbox v1.9 software (GE Healthcare Biosciences, Piscataway, NJ). For the phospho$^{(S345)}$- and total Chk1 assays: the raw data values (fluorescent nuclear intensity for PBMCs), the percentage change for phospho$^{(S345)}$ biomarker was calculated for each subject by comparing the levels measured at each post-dose time point to pre-dose levels measured at baseline. The number of fluorescent γH2AX foci within individual PBMC cells were quantified using the IN Cell Investigator Developer Toolbox v1.9 software and the percentage of cells at each time point with greater than 5 foci calculated. The assays were GCP compliant and utilized healthy volunteer PBMCs as quality controls in every analytical run.

Flow cytometry

Peripheral blood was drawn into 8 mL EDTA tubes (Vacutainer, BD) and analyzed within 24 hours. ACK-lysed whole blood (for myeloid panel) or PBMC from density gradient centrifugation (for lymphocyte panel) were used. Samples were surface-stained with antibodies for 30 mins at

4 °C. Samples were analyzed on a LSR Fortessa (BD Biosciences). FACS analyses were performed in FlowJo v10.

Antibodies used

Mix 1

NKG2D	BV421	Biolegend
CD4	BV510	Biolegend
CD45	BV650	Biolegend
TCRγδ	FITC	Biolegend
CD25	PE	Biolegend
CD56	PE-Dazzle 594	Biolegend
CD73	APC	Biolegend
CD62L	PerCp Cy5.5	Biolegend
CD3	AF700	Biolegend
CD8	Pe-Cy7	Biolegend
CD127	APC-Cy7	Biolegend

Mix 2:

NKp46	BV421	Biolegend
CD4	BV510	Biolegend
CD45	BV650	Biolegend
TCRγδ	FITC	Biolegend
OX-40	PE	Biolegend
CD56	PE-Dazzle 594	Biolegend
4-1BB	APC	Biolegend
NKp30	PerCp Cy5.5	Biolegend
CD3	AF700	Biolegend
CD8	Pe-Cy7	Biolegend
CD69	APC-Cy7	Biolegend

Mix 3:

NKG2A	BV421	BD
CD4	BV510	Biolegend
CD45	BV650	Biolegend
TCRγδ	FITC	Biolegend
PD-1	PE	Biolegend
CD56	PE-Dazzle 594	Biolegend
LAG-3	APC	R&D Systems
TIGIT	PerCp Cy5.5	eBioscience
CD3	AF700	Biolegend
CD8	Pe-Cy7	Biolegend
TIM-3	APC-Cy7	Biolegend

Mix 4:

CD45RO	Pacific Blue	Biolegend
CD4	BV510	Biolegend
CD45	BV650	Biolegend
CD45RA	FITC	Biolegend
CCR7	PE	Biolegend
CD56	PE-Dazzle 594	Biolegend
CD27	APC	Biolegend
CD62L	PerCp Cy5.5	Biolegend
CD3	AF700	Biolegend
CD8	Pe-Cy7	Biolegend
CD16	APC-Cy7	Biolegend

Mix 5:

CD56	BV421	Biolegend
CD4	BV510	Biolegend
CD45	BV650	Biolegend
TCRγδ	FITC	Biolegend
LAP	PE	Biolegend
CTLA-4	PE-Dazzle 594	Biolegend
ICOS	APC	Biolegend
CD57	PerCp Cy5.5	Biolegend
CD3	AF700	Biolegend
CD8	Pe-Cy7	Biolegend
CD95	APC-Cy7	Biolegend

Mix 6:

PVR	BV421	Biolegend
CD19	BV510	Biolegend
CD45	BV650	Biolegend
CD3	FITC	Biolegend
PD-L1	PE	Biolegend
CD38	PE-Dazzle 594	Biolegend
CD27	APC	Biolegend
CD62L	PerCp Cy5.5	Biolegend
CD14	AF700	Biolegend
HLA-E	Pe-Cy7	Biolegend
CD56	APC-Cy7	Biolegend

Mix 7:

NKG2A	BV421	BD
CD8	BV510	Biolegend
CD45	BV650	Biolegend
CD69	FITC	Biolegend
PD-1	PE	Biolegend
CD38	PE-Dazzle 594	Biolegend
CD56	APC	Biolegend
TIGIT	PerCp Cy5.5	eBioscience
CD3	AF700	Biolegend
CD57	Pe-Cy7	Biolegend
CD16	APC-Cy7	Biolegend

MDSC/DC/monocytes

CD33	P67.6	APC		Biolegend
CD11b	ICRF44	PE		Biolegend
CD11c	3.9	BV421		Biolegend
CD15	HI98	FITC		Biolegend
HLA-DR	L243	PerCP Cy5.5		Biolegend
CD14	M5E2	BV510		Biolegend
CCR2	K036C2	Pe-Cy7		Biolegend
CD16	3G8	APC-C7		Biolegend
Lin (CD3, CD19, CD56)				
CD45	HI30	BV650		Biolegend
Isotype	MOPC-173	AF700		

Gating strategy

See supp Fig. 13, 14

Plasma cytokine analysis

Plasma was taken from EDTA tubes (BD vacutainer) used for buffy coat or immune profiling samples. Plasma was removed, re-spun and aliquoted into 1.5 mL tubes and stored at -70 ^{o}C until analysis. Samples were run in duplicate using the Bio-Plex Pro Human Cytokine 27-plex panel (Bio-Rad) as per manufacturer's instructions.

Criteria for Dose Limiting Toxicity

1. Hematological toxicity
    1. Grade 3 thrombocytopenia with bleeding
    2. Grade 4 thrombocytopenia
    3. Grade 4 neutropenia lasting >7 days in the absence of growth factor support
    4. Grade 4 neutropenia of any duration accompanied by fever ≥ 38.5°C and/or systemic infection
    5. Any other grade ≥4 hematological toxicity
2. Cardiovascular toxicity
    1. Clinically significant hypotension defined as an asymptomatic fall in systolic blood pressure more than 20mmHg to below 70mmHg persisting for at least 10 minutes
    2. Symptomatic orthostatic fall in systolic blood pressure of more than 20mmHg compared to resting supine blood pressure
    3. Prolongation of QTc >0.5 seconds (using an appropriate correction QTcB (Bazzett) or QTcF (Fridericia))
3. Gastrointestinal/hepatic toxicity
    1. ALT or AST >5 times ULN; ALP >5 times ULN with elevated gamma- GT

    2. ALT or AST >3 times ULN (or ALP >3 times ULN with elevated gamma-GT) with the appearance of symptoms associated with a clinical diagnosis of hepatitis including right upper quadrant pain or tenderness, fever, rash, or eosinophilia (>5%)
    3. [ALT or AST >3 times ULN] and [total bilirubin >2 times ULN] or INR >1.5x ULN (unless patient receiving warfarin) (or other evidence of impaired liver synthetic function)
4. Any other grade 3-4 non-hematological toxicity occurring during the time period from the first dose to the end of the first cycle
    1. Excluding grade 3 diarrhea which resolves to grade 1 within 24 hours of medical management
    2. Excluding grade 3 nausea or vomiting if ameliorated by medical management
    3. Excluding grade 3 fatigue unless there is an increase by 2 grades from baseline
5. Any dose in cycle 1, or the start of cycle 2, delayed by more than 7 days due to toxicity or patient unable to complete cycle 1 at the planned dose due to toxicity (parts A and B only)
6. Any other toxicity that is greater than baseline, is clinically significant and/or unacceptable, does not respond to supportive care, results in a disruption of dosing schedule of more than 14 days or is considered to be dose-limiting by the investigators.

List of supplementary figures and tables

Supp Fig1: Bone marrow toxicity, by dose and schedule

Supp Fig 2: Accumulation ratios

Supp Fig 3: Change in PBMC phospho-(S345)Chk1 fluorescence intensity after 2-week treatment

Supp Fig 4: Tumor kinetics for participants at 1 site (Royal Marsden).

Supp Fig 5: Duration on study by mutational status.

Supp Fig 6: Oncoprint of more detailed mutational information, sorted by duration on study.

Supp Fig 7: Tumour mutational burden, by clinical benefit

Supp Fig 8A, B: Heatmap of top 100 differentially expressed genes, in baseline biopsies

Supp Fig 9: Geneset enrichment analysis using the 'Hallmarks' gene set.

Supp Fig 10: CIBERSORTx cell-type deconvolution data, by response

Supp Fig 11: cell-type signatures.

A: T-cell functions signature, baseline biopsies

B: NK cell function signature, baseline biopsies

C: cytokine signature, on-treatment biopsies

D: T-cell functions signature, on-treatment biopsies

Supp Fig 12: Interferon-stimulated gene signature.

Supp Fig 13: Flow cytometry gating strategy for monocytes, MDSC, and DC

Supp Fig 14: Lymphocyte gating strategy

Supp Table 1: SAE related to ceralasertib (judged by investigator as definitely, probably or possibly related).

Supp Table 2: Dose-limiting toxicities

Supp Table 3: Genes of interest in sequencing panel, used for selection.

Supp table 4: SWI/SNF mutations

Supplementary References

47. *Guidance for Industry, Bioanalytical Method Validation, U.S. Department of Health and Human Services, Food and Drug Administration, Center for Drug Evaluation and Research (CDER), Center for Veterinary Medicine (CMV)*. Rockville, MD: Food and Drug Administration; 2018.
48. Subramanian A, Tamayo P, Mootha VK, Mukherjee S, Ebert BL, Gillette MA, et al. Gene set enrichment analysis: a knowledge-based approach for interpreting genome-wide expression profiles. *Proceedings of the National Academy of Sciences of the United States of America.* 2005;102(43):15545-50.
49. Liberzon A, Birger C, Thorvaldsdóttir H, Ghandi M, Mesirov JP, and Tamayo P. The Molecular Signatures Database (MSigDB) hallmark gene set collection. *Cell Syst.* 2015;1(6):417-25.
50. Bindea G, Mlecnik B, Tosolini M, Kirilovsky A, Waldner M, Obenauf AC, et al. Spatiotemporal dynamics of intratumoral immune cells reveal the immune landscape in human cancer. *Immunity.* 2013;39(4):782-95.
51. Newman AM, Steen CB, Liu CL, Gentles AJ, Chaudhuri AA, Scherer F, et al. Determining cell type abundance and expression from bulk tissues with digital cytometry. *Nat Biotechnol.* 2019;37(7):773-82.
52. Jones GN, Rooney C, Griffin N, Roudier M, Young LA, Garcia-Trinidad A, et al. pRAD50: a novel and clinically applicable pharmacodynamic biomarker of both ATM and ATR inhibition identified using mass spectrometry and immunohistochemistry. *British journal of cancer.* 2018.
53. Bankhead P, Loughrey MB, Fernández JA, Dombrowski Y, McArt DG, Dunne PD, et al. QuPath: Open source software for digital pathology image analysis. *Scientific Reports.* 2017;7(1):16878.
54. Schmidt U, Weigert M, Broaddus C, and Myers G. In: Frangi AF, Schnabel JA, Davatzikos C, Alberola-López C, and Fichtinger G eds. *Medical Image Computing and Computer Assisted Intervention – MICCAI 2018*. Cham: Springer International Publishing; 2018:265-73.

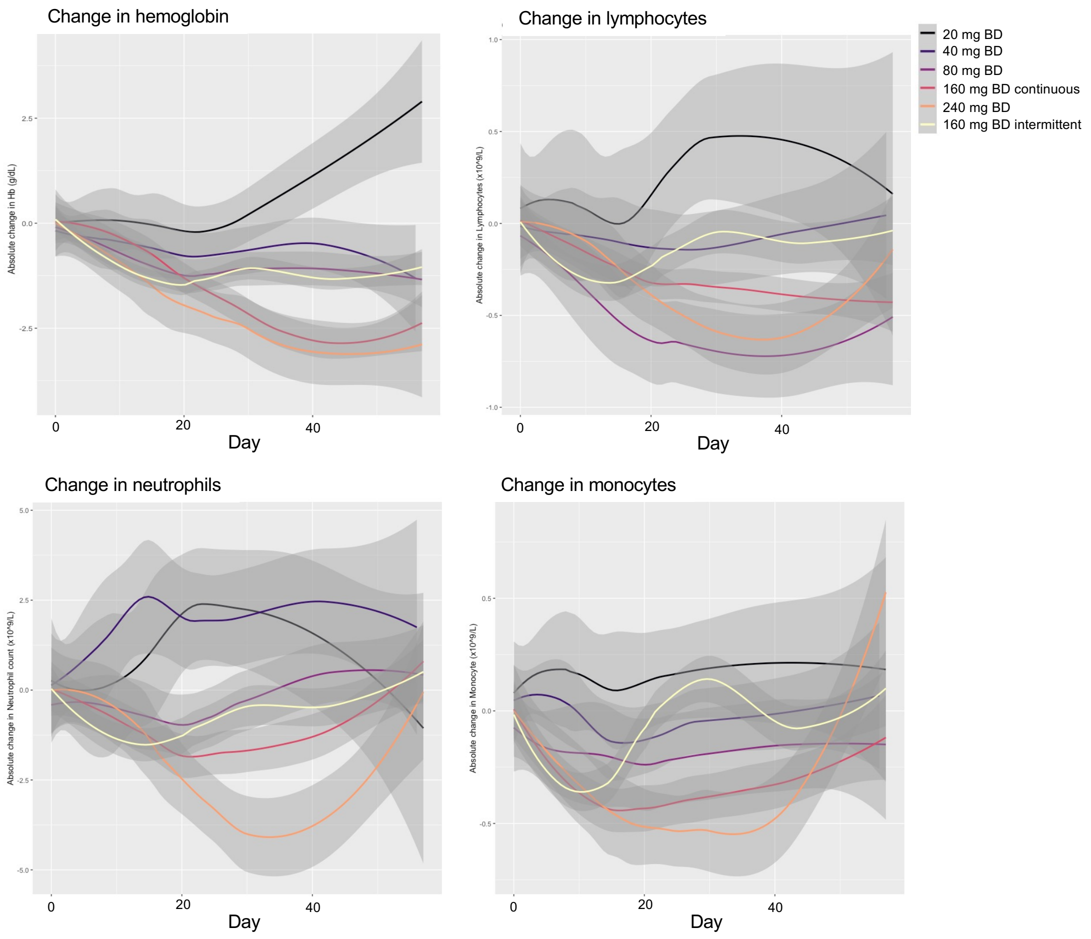

Supp Fig 1
Bone marrow toxicity, by dose and schedule
Change in full blood count parameters with time, by dose cohort. Smoothed conditional mean absolute changes compared with baseline blood count are presented, with 95% CI.

CTCAE term	240 mg BD	160 mg BD continuous	160mg BD Intermittent	80 mg BD
Anemia	1, G4	1, G2	1, G3	1, G3
Platelet count decreased	2, G4			
Blood bilirubin increased			1, G3	
Increased amylase	1, G3			
Non-cardiac chest pain		1, G3		
Urinary tract infection		1, G3		
Electrocardiogram QTc interval prolonged		1, G1		
Syncope			1, G3	1, G3
Left ventricular systolic dysfunction		1, G3		
Pneumonitis	1, G2			
Photosensitivity	1, G3			

Supp Table 1
SAE related to ceralasertib (judged by investigator as definitely, probably or possibly related). No related SAE at 20 and 40 mg dose levels

Dose cohort	Number in cohort evaluable for DLT	DLT	Number of DLT
20 mg BD	3	none	0
40 mg BD	3	none	0
80 mg BD	6	Thrombocytopenia, G3 with epistaxis (n=1)	1
160 mg BD	6	none	0
240 mg BD	6	Increased amylase, G3 (n=1), thrombocytopenia G4 (n=2)	3

Supp Table 2
Dose-limiting toxicities

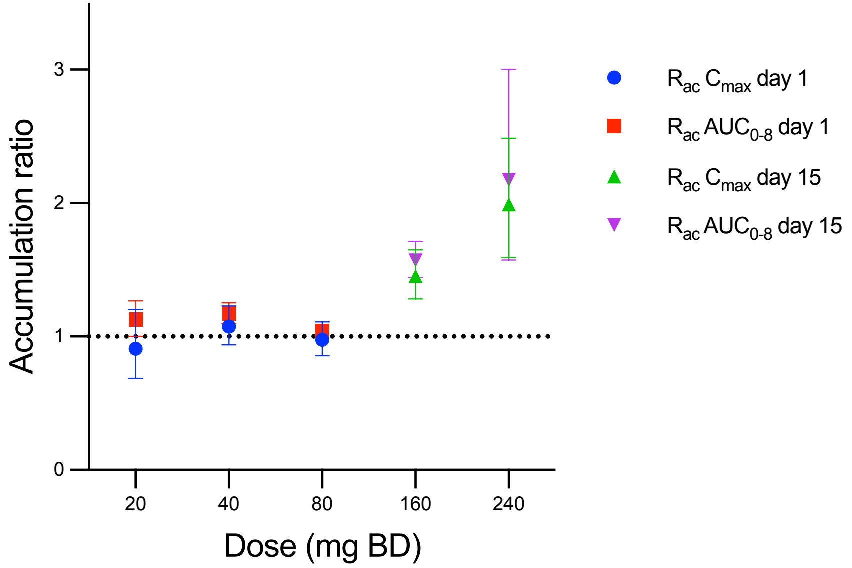

Supp Fig 2
Accumulation ratios
accumulation ratio of ceralasertib, calculated by geometric mean Cmax or AUC0-8 at day 1 vs. day 0, or day 15 vs. day 0 for the indicated dose levels.

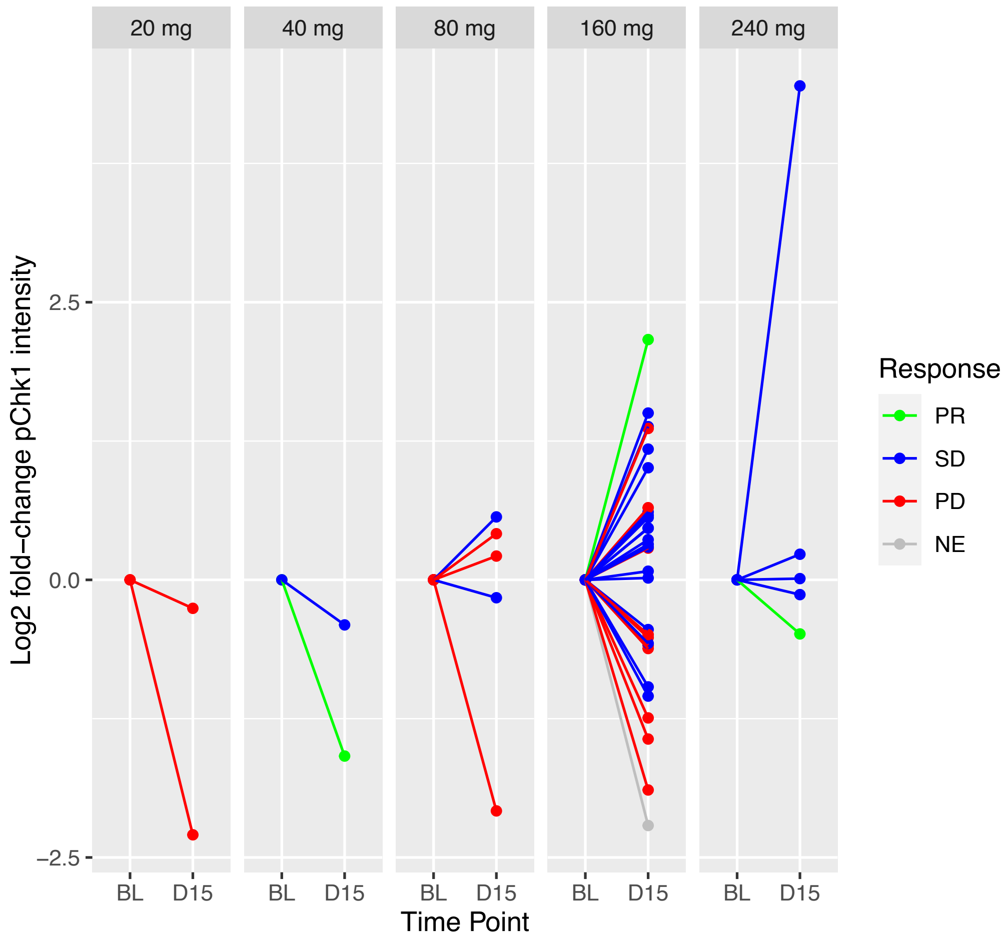

Supp Fig 3
Change in PBMC phospho-(S345)Chk1 fluorescence intensity after 2-week treatment (D15), normalized to baseline (BL) per patient, by dose level. Color of line indicates RECIST response.

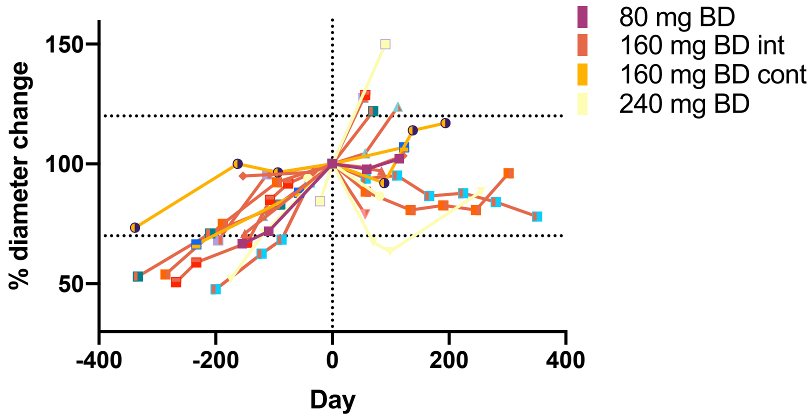

Supp Fig 4: tumor kinetics for participants at 1 site (Royal Marsden). Medical records were reviewed to obtain prior tumor measurements (negative days) for the target lesions. These were taken and normalized to the baseline measurements for this study (day 0 measurement).

ATM-G1	ATR	CIN	DSB	EXCISION	FANC	HR	MMR
ATM*	ATR	AURKA	CDK12	LIG1	C17orf70 / FA	BABAM1	MLH1
CDKN1A (p21	ATRIP	POLD1*	H2AFX	LIG4	DNA2	BARD1	MLH3
CDKN2A (p16	ATRX	POLD2	RNF168		FANCA	BRCA1	MSH2
CDKN2B	CDC25A	POLD3	SLX4*	**RECOVERY**	FANCB	BRCA2	MSH3
CHEK2	CDC25C	POLD4		BLM	FANCC	BRIP1	MSH6
KAT5	CHEK1	POLE		EME1	FANCD2	DCLRE1C	PMS2
MDC1	CLSPN	PTEN		MUS81	FANCE	EXO1	RECQL
MDM2	HUS1			WRN	FANCF	GEN1	
MDM4	RAD1	**NER**	**NHEJ**	**SWI/SNF**	FANCG	PALB2*	**ONCOGENE**
MRE11A	RAD17	ERCC1*	SETMAR	ARID1A*	FANCI	PARP1*	BRAF
NBN	RAD9A	ERCC4*	TP53BP1*	ARID1B	FANCL	PARP2	CCND1
PRKDC	RBBP8	XRCC1*	XRCC5	ARID2	FANCM	Rad51	CCNE1*
RAD50	RPA1		XRCC6	SMARCA4	XRCC2	RAD51AP1	HRAS
TP53	TOPBP1			SMARCB1		RAD51B	KRAS
						RAD51C	MYC*
					**TLS**	RAD51D	MYCL*
					MAD2L2	RAD54B	MYCN*
					REV3L*	RAD54L	NRAS
						XRCC3	
						XRCC4	

Supp Table 3: genes of interest in sequencing panel, used for selection. * Signifies those with published data to support ATRi sensitization

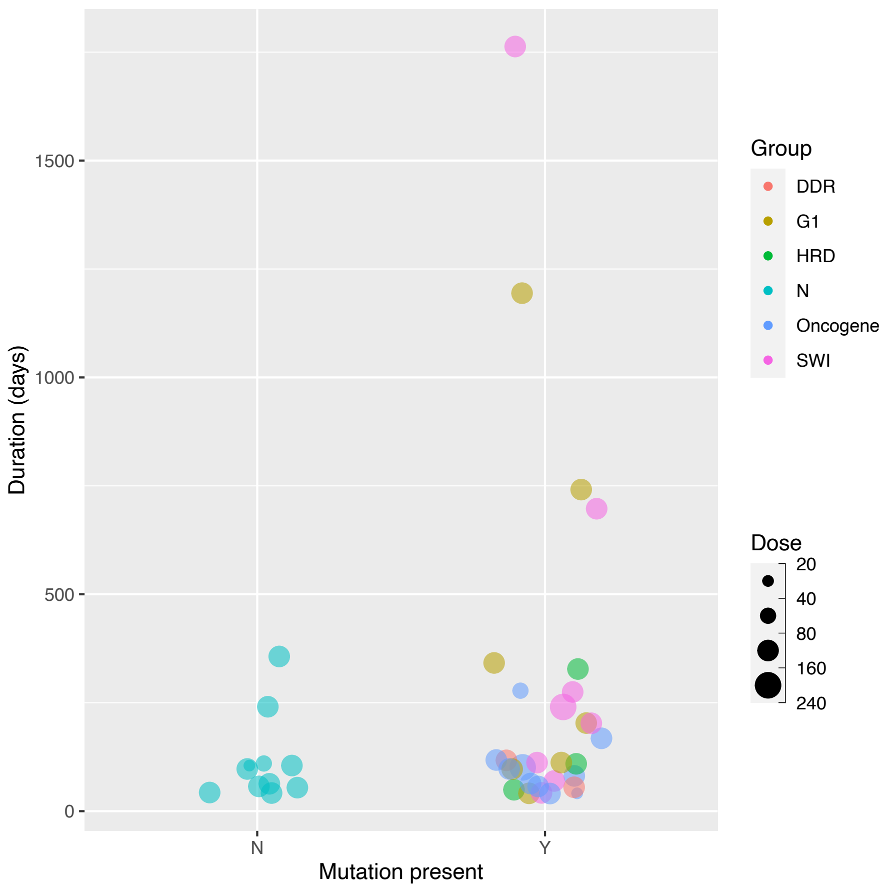

Supp Fig 5
Duration on study by mutational status. DDR: DNA damage response defect (not otherwise mentioned); G1: ATM -G1 pathway; HRD: homologous recombination pathway; Oncogene: driver mutation; SWI: SWI/SNF pathway mutation; N = no mutation. Participants who had available sequencing data for their tumors were included. If sequencing was not done, they were not included. Mutation of interest defined in Supp. Table 3.

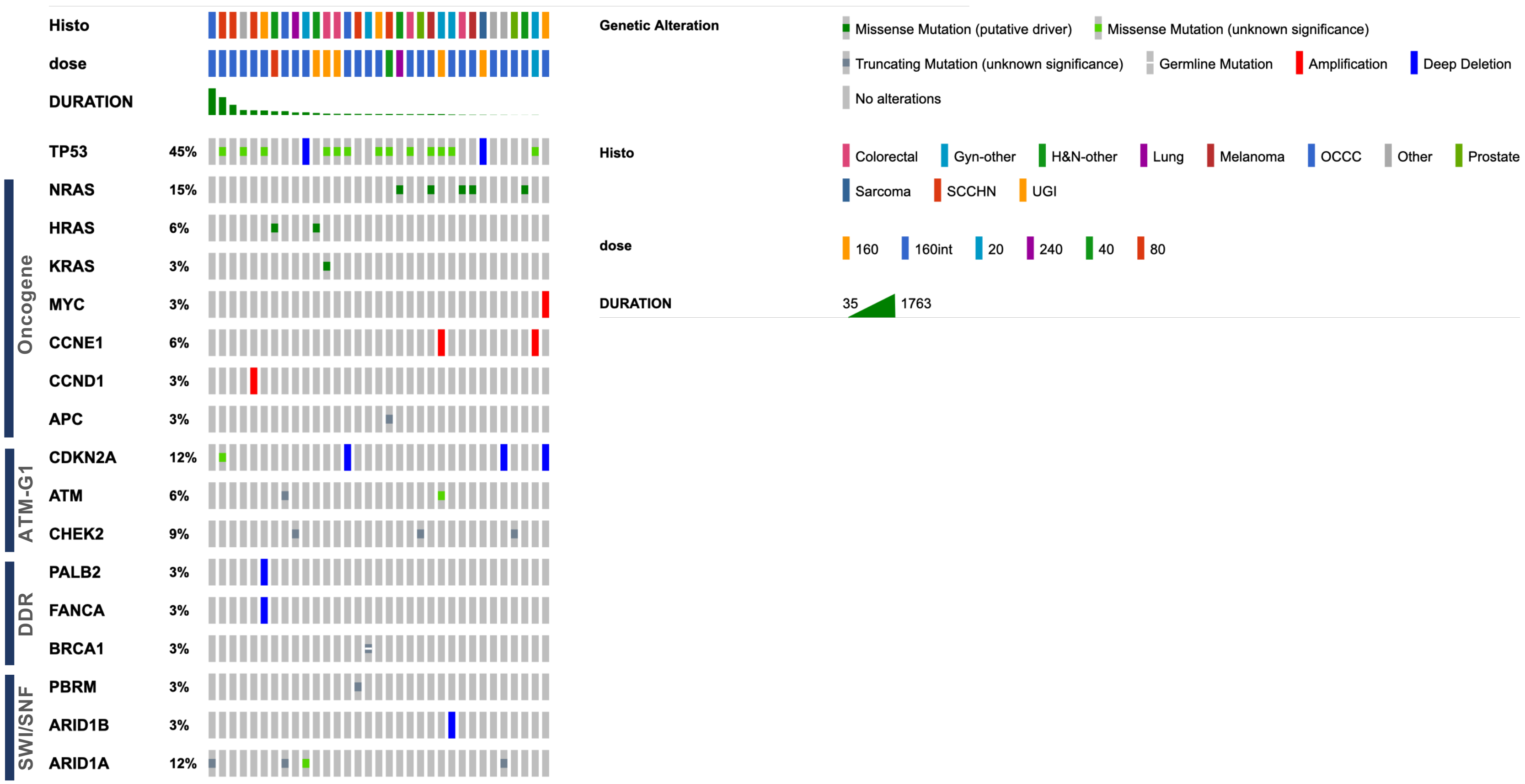

Supp Fig 6
Oncoprint of more detailed mutational information, sorted by duration on study. Oncoprint generated using cBioPortal [44].

Gene	Mutation	Mutation type	Protein loss	Histology	Response	Duration (d)	Coexisting mutation	TMB (mut/Mb)
**ARID1A**	E1763fs	Frameshift	Yes (H 0)	Ovarian clear cell (Fig. 3J)	PR	1763	PI3KCA missense	70.97
**ARID1A**	R693*	Truncating	Yes (H 0)	Eccrine adenocarcinoma (Fig. 3K)	SD	240		13.33
**ARID1A**	S1623L	Missense	No (H 300)	Cervix adenocarcinoma (Fig. 3M)	SD	203	SMARCA4 loss, cyclin E overexpression	9.37
**ARID1A**	Q1188X	Missense	No (H 235)	Ovarian clear cell (Fig. 3N)	SD	275	ATM frameshift, 50% ATM+ (protein)	12.05
**ARID1A**	E1387X	Missense	No (H 290)	Lobular breast carcinoma (Fig. 3L)	PD	42	CDKN2A loss	18.75
**PBRM**	S295*	Truncating	unknown	SCCHN	SD	112		5.36
**ARID2**	Y854fs	Frameshift	Unknown	SCCHN	SD	697		10.71

Supp table 4
SWI/SNF mutations
TMB: tumor mutational burden.

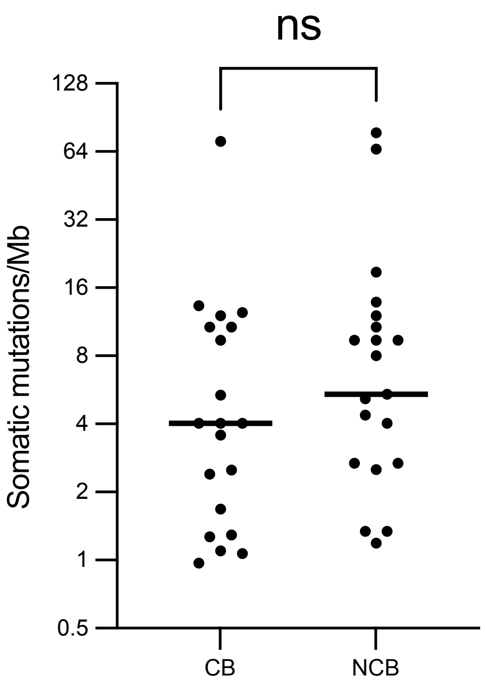

Supp Fig 7
Tumor mutational burden, where available.
CB: clinical benefit, defined as best response of RECIST PR or RECIST SD for 112 days or more
NCB: no clinical benefit, defined as best response of RECIST PD or RECIST SD for less than 112 days.

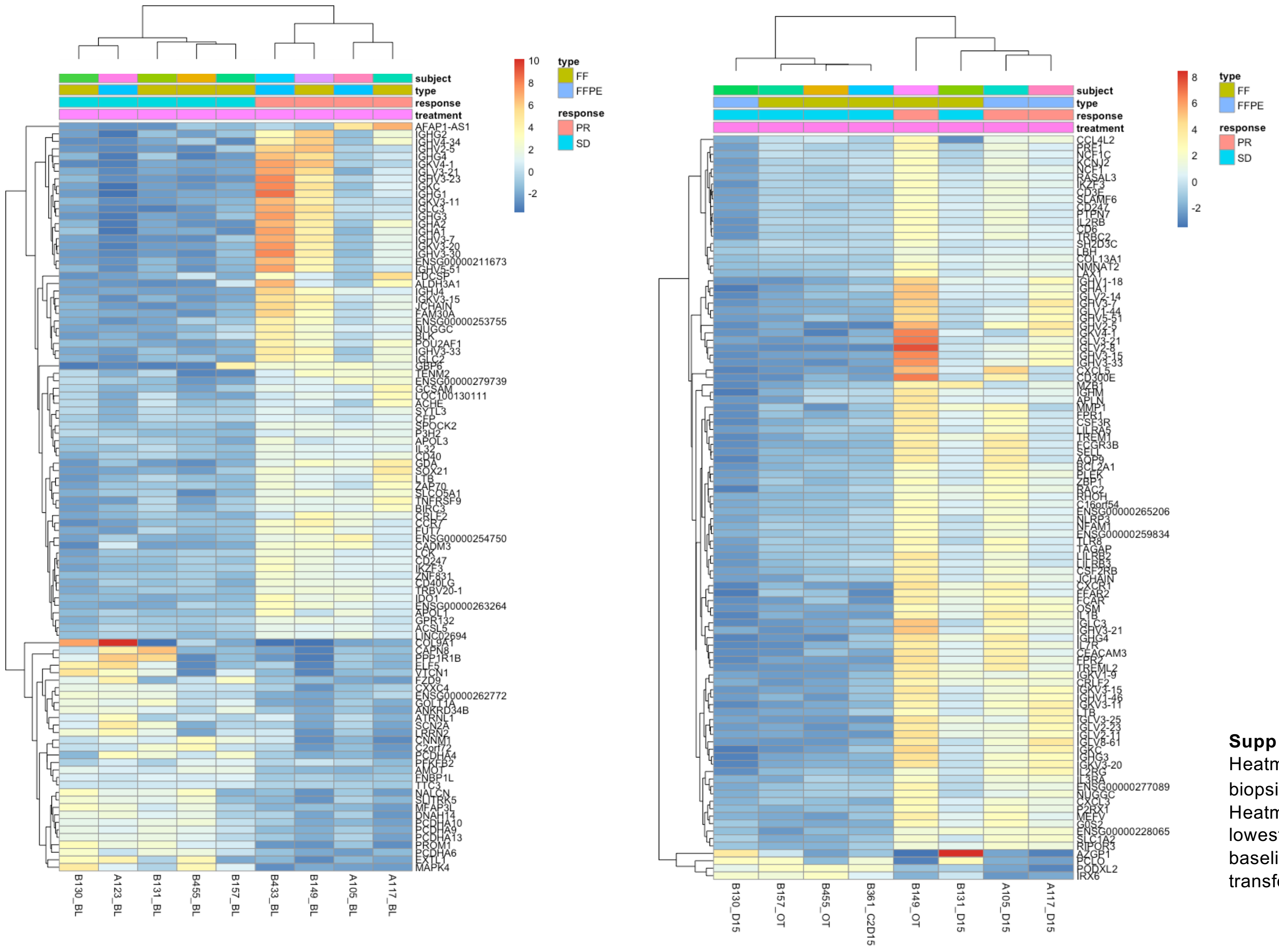

Supp Fig 8A (left) B (right)

Heatmap of top 100 differentially expressed genes, in baseline biopsies

Heatmap of top 100 differentially expressed genes with the lowest adjusted p-value, clustering according to response for (A) baseline and (B) on-treatment tumor biopsies. The data are rlog transformed.

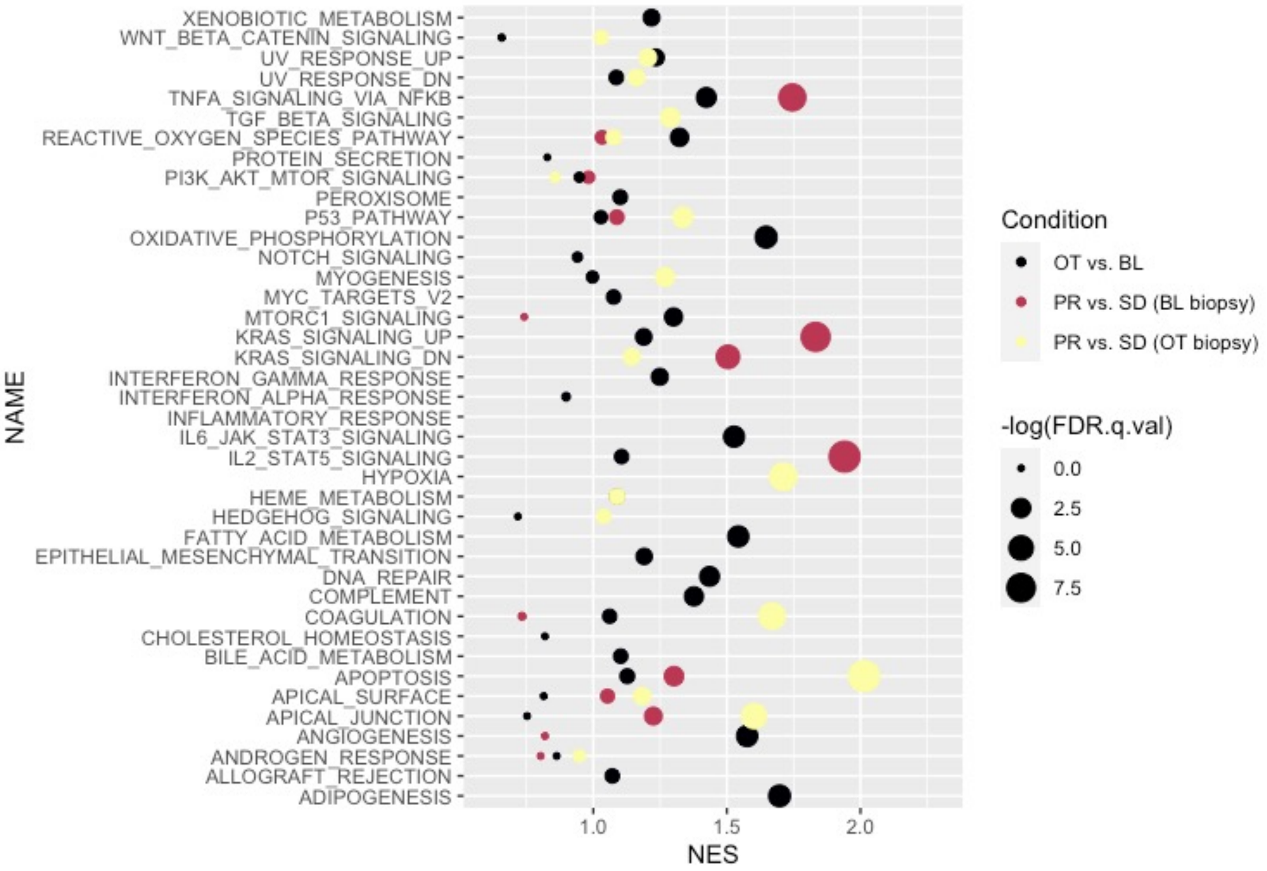

Supp Fig 9
Geneset enrichment analysis using the 'Hallmarks' gene set.

Pathway analysis of differentially expressed genes. Gene set enrichment analysis was carried out using the 'hallmarks' gene set. Normalized expression data for each condition was used from DEseq2. Conditions: OT vs. BL: all samples, on-treatment vs. baseline biopsy; PR vs. SD (BL biopsy): differences between patients having RECIST partial response (PR) or stable disease (SD) i.e., responders vs non-responders, comparing baseline biopsies; PR vs. SD (OT biopsy) difference between responders and non-responders, comparing on-treatment biopsies. Color indicates condition, size indicates false discovery rate q-value, 'NES': normalized enrichment score.

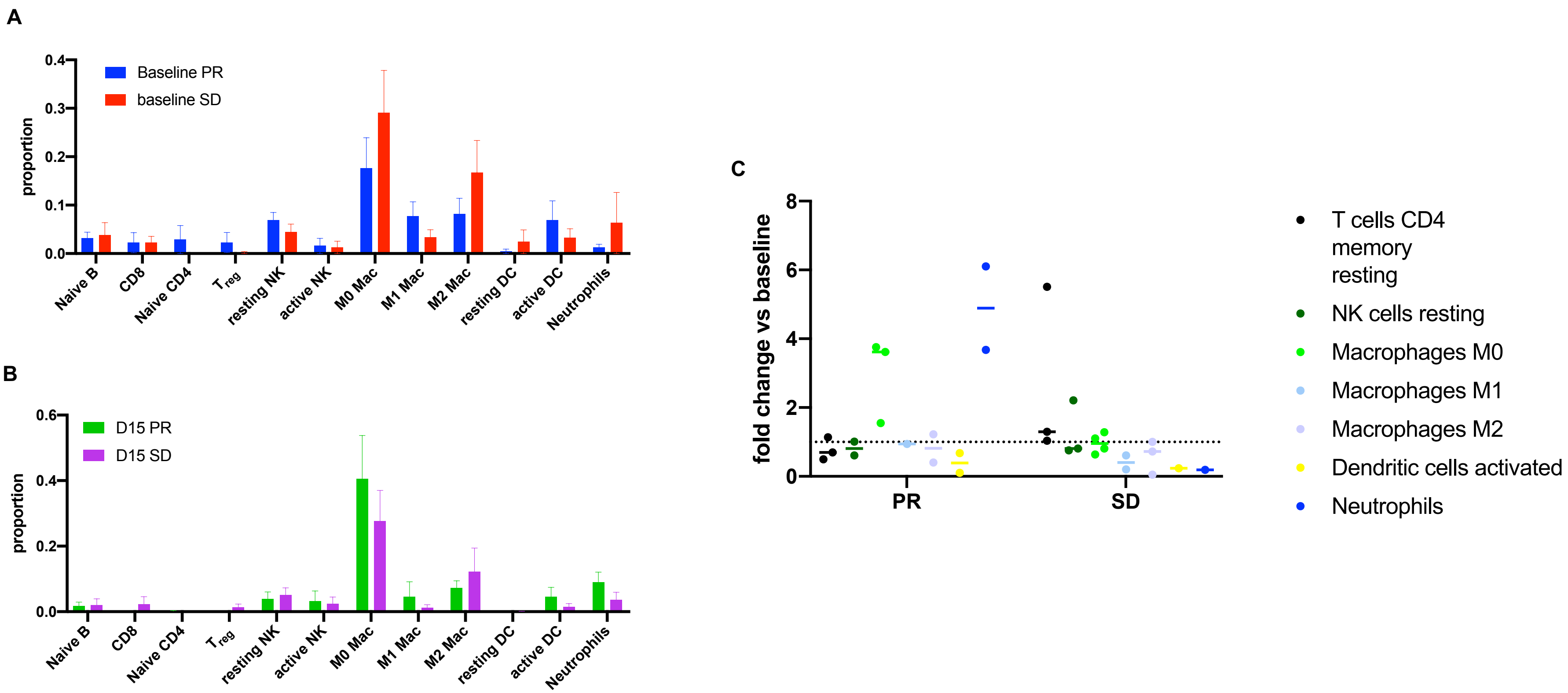

Supp Fig 10
CIBERSORTx cell-type deconvolution data, by response
A: Baseline samples, split by RECIST response
B: On-treatment samples, split by RECIST response
C: Fold-change in cell type proportion (on-treatment divided by baseline) split by RECIST response

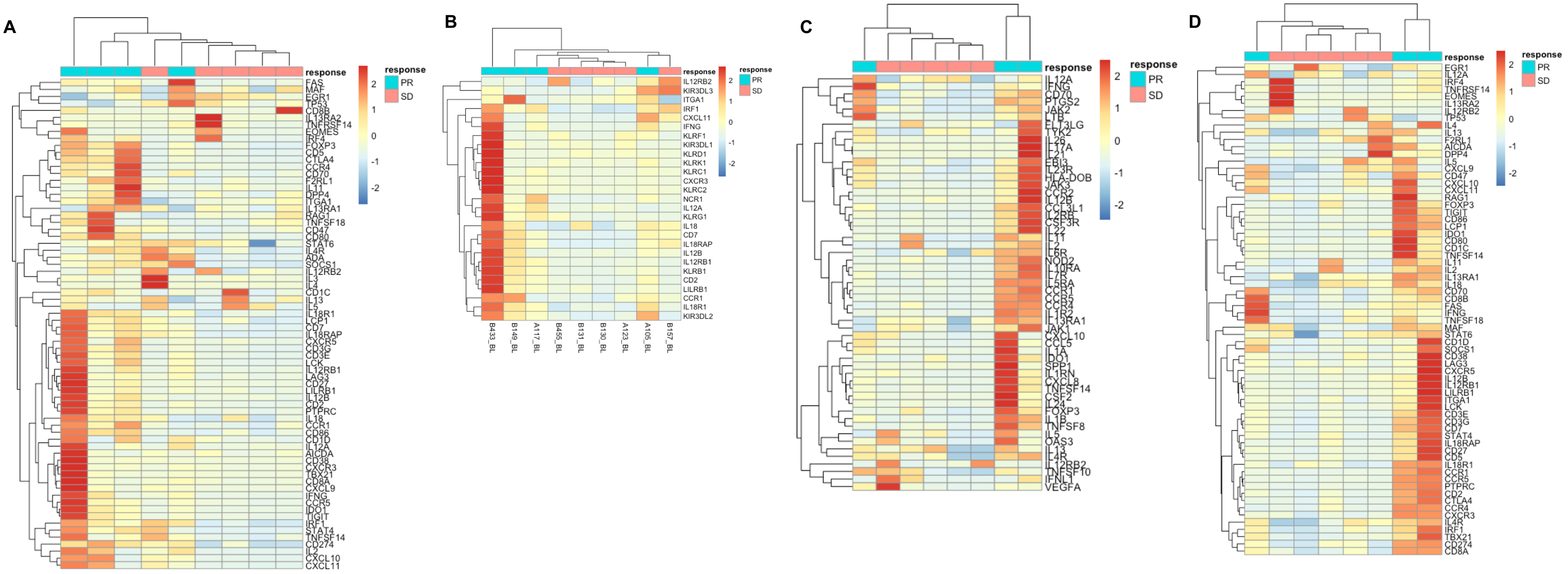

Supp Fig 11: cell-type signatures.
A: T-cell functions signature, baseline biopsies
B: NK cell function signature, baseline biopsies
C: cytokine signature, on-treatment biopsies
D: T-cell functions signature, on-treatment biopsies

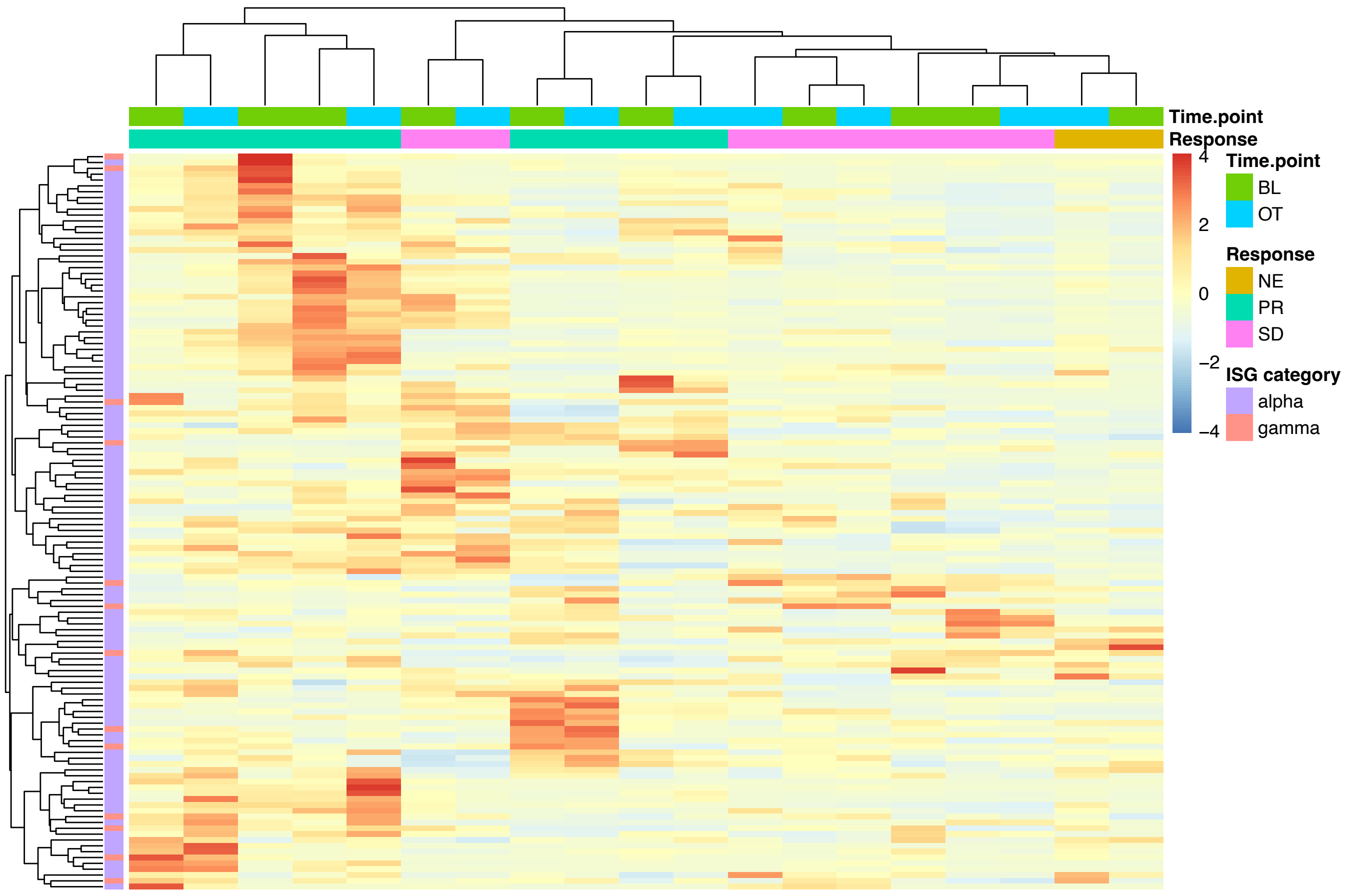

Supp Fig 12: Interferon-stimulated gene signature. Interferon-stimulated genes, and categorisation by alpha or gamma interferon stimulation were obtained from Liu et al., 2011, PNAS 109(11): 4239. "NE" indicates a patient who was not evaluable for response assessment. "BL" baseline biopsy, "OT" on-treatment biopsy (generally day 15)

Supp Fig 13
flow cytometry gating strategy for monocytes, MDSC, and DC

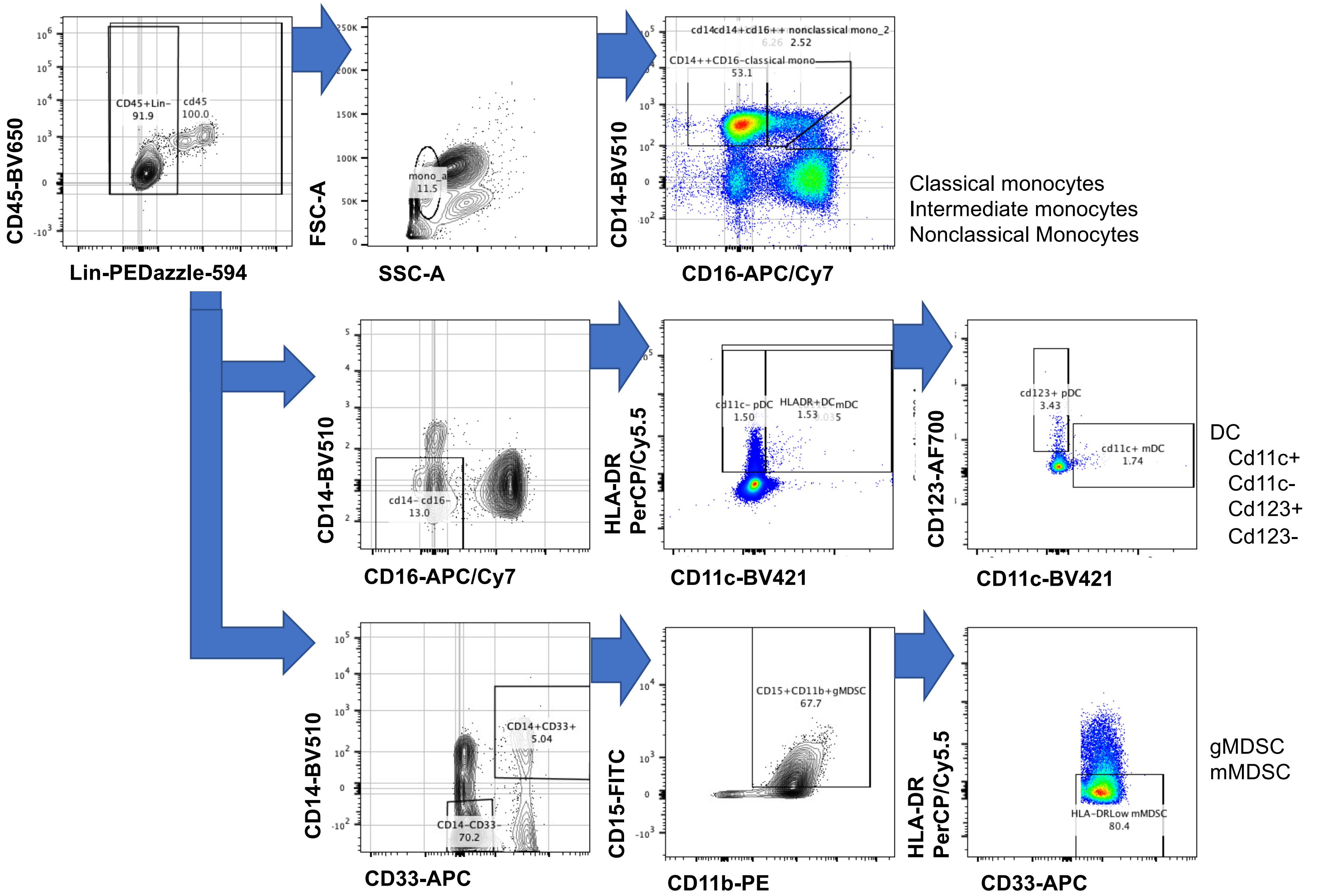

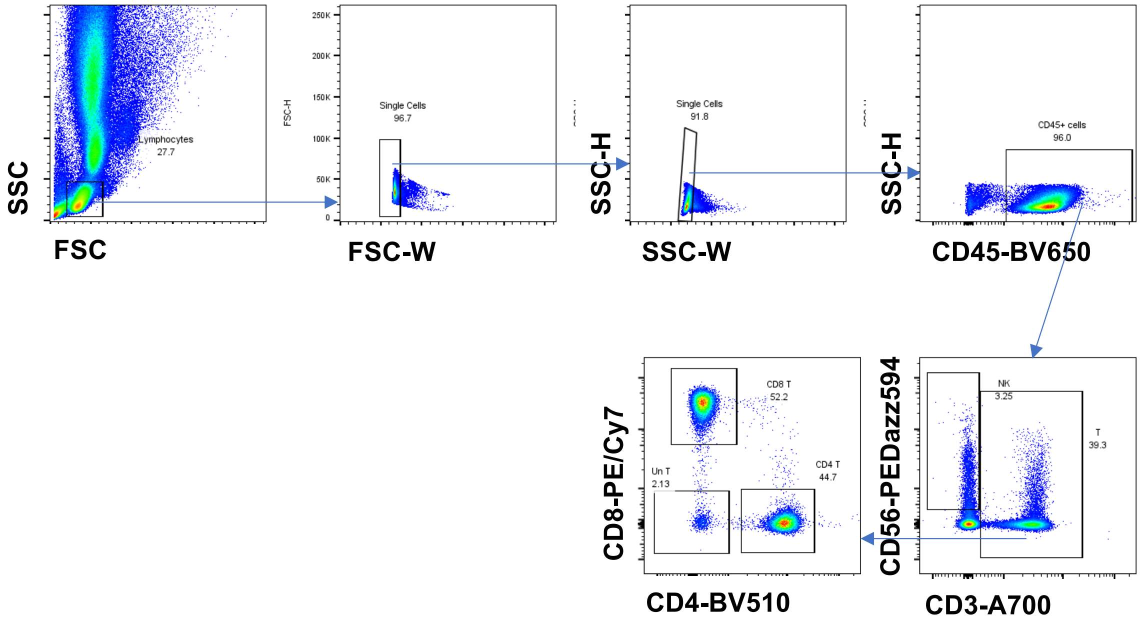

Supp Fig 14
Lymphocyte gating strategy
